# Synergy of Solid-State NMR, Single-Crystal X-ray Diffraction, and Crystal Structure Prediction Methods: A Case Study of Teriflunomide (TFM)

**DOI:** 10.1021/acs.cgd.1c00123

**Published:** 2021-05-10

**Authors:** Tomasz Pawlak, Isaac Sudgen, Grzegorz Bujacz, Dinu Iuga, Steven P. Brown, Marek J. Potrzebowski

**Affiliations:** † Centre of Molecular and Macromolecular Studies, Polish Academy of Sciences, Sienkiewicza 112, 90-363 Lodz, Poland; ‡ Molecular Systems Engineering Group, Centre for Process Systems Engineering, Department of Chemical Engineering, Imperial College London, London SW7 2AZ, U.K.; § Institute of Molecular and Industrial Biotechnology, Lodz University of Technology, Stefanowskiego 4/10, 90-924, Lodz, Poland; ∥ Department of Physics, University of Warwick, Coventry CV4 7AL, U.K.

## Abstract

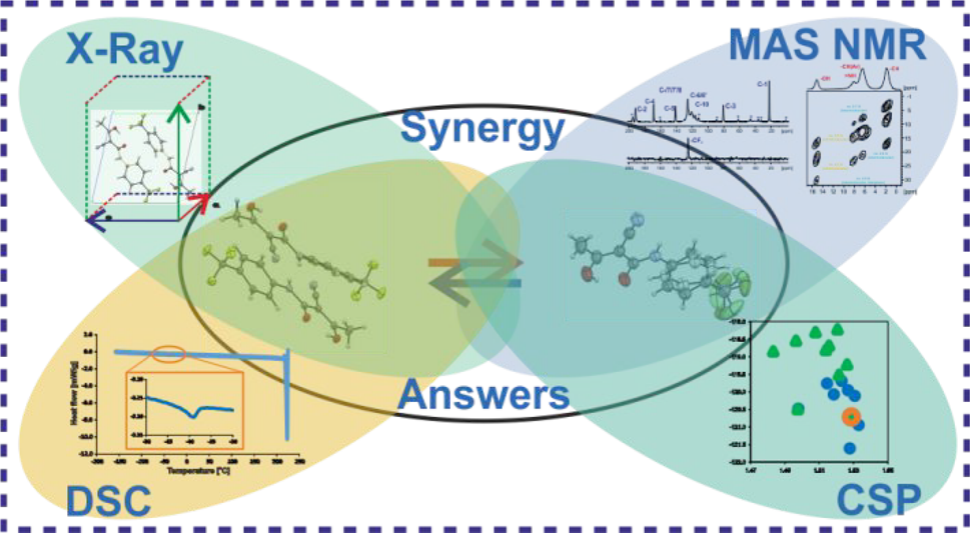

In this work, for the first time, we present the X-ray diffraction crystal structure and spectral properties of a new, room-temperature polymorph of teriflunomide (TFM), CSD code 1969989. As revealed by DSC, the low-temperature TFM polymorph recently reported by Gunnam et al. undergoes a reversible thermal transition at −40 °C. This reversible process is related to a change in *Z’* value, from 2 to 1, as observed by variable-temperature ^1^H–^13^C cross-polarization (CP) magic-angle spinning (MAS) solid-state NMR, while the crystallographic system is preserved (triclinic). Two-dimensional ^13^C–^1^H and ^1^H–^1^H double-quantum MAS NMR spectra are consistent with the new room-temperature structure, including comparison with GIPAW (gauge-including projector augmented waves) calculated NMR chemical shifts. A crystal structure prediction procedure found both experimental teriflunomide polymorphs in the energetic global minimum region. Differences between the polymorphs are seen for the torsional angle describing the orientation of the phenyl ring relative to the planarity of the TFM molecule. In the low-temperature structure, there are two torsion angles of 4.5 and 31.9° for the two *Z’* = 2 molecules, while in the room-temperature structure, there is disorder that is modeled with ∼50% occupancy between torsion angles of −7.8 and 28.6°. These observations are consistent with a broad energy minimum as revealed by DFT calculations. PISEMA solid-state NMR experiments show a reduction in the C–H dipolar coupling in comparison to the static limit for the aromatic CH moieties of 75% and 51% at 20 and 40 °C, respectively, that is indicative of ring flips at the higher temperature. Our study shows the power of combining experiments, namely DSC, X-ray diffraction, and MAS NMR, with DFT calculations and CSP to probe and understand the solid-state landscape, and in particular the role of dynamics, for pharmaceutical molecules.

## Abstract 2

This work presents the X-ray diffraction crystal structure and spectral properties of a new, room-temperature polymorph of teriflunomide that undergoes a reversible thermal transition, and it is related to the change in *Z*′ value, from 2 to 1. Our study shows the power of combining experiments with DFT calculations and CSP to understand the solid-state landscape for pharmaceutical molecules.

## 1 Introduction

One particular scientific method is usually not enough to solve a challenging problem relating to solid matter for organic molecules, such as a moderately sized active pharmaceutical ingredient (API) organic molecule. Understanding the complexity of such solid matter which depends on the interplay of a number of subtle, differentiated intermolecular contacts requires the application of different diagnostic tools and should be supported by theoretical methods. For crystalline compounds, X-ray diffraction and solid-state NMR are usually the experimental techniques of choice. In the group of theoretical methods, crystal structure prediction (CSP) developed during the last two decades has become one of the most promising approaches.^1−5^ CSP methods are based on searching for the most thermodynamically stable crystal structure, making various approximations in evaluating the crystal energy. The most stable (global minimum) structure provides a prediction of an experimental crystal structure. Several successful CSP studies on large but relatively rigid systems, such as organic porous cages, have been reported.^6,7^ The *ab initio* random structure search (AIRSS) method that has been used successfully for a number of inorganic solids^8−11^ has also been applied to a simple organic molecule.^12^ Organic compounds are more challenging, since they tend to have a considerable conformational flexibility and can crystallize in a variety of conformations.^13^ In such cases, the CSP search space grows exponentially and the prediction of experimentally observed crystal structures becomes ever more challenging. The support of this process by experimental techniques such as solid-state magic-angle spinning (MAS) NMR spectroscopy^14,15^ can significantly reduce the complexity of the problem.

Solid-state MAS NMR spectroscopy is a powerful method for characterizing polymorphs, solvates, salts, and cocrystals exhibited by organic molecules.^16^ The power of this technique is also due to the fact that, using different NMR experiments, subtle structural features as well as dynamic properties that report on the time scale and amplitude of motion can be measured. Solid-state NMR spectroscopy is very sensitive to local molecular disorder (including dynamic disorder) and structural defects. Thus, it is no surprise that this method is frequently used in different branches of science, in particular in the pharmaceutical sciences, to study structural features and to verify the quality and/or homogeneity of the studied material.^17−39^

As noted above, there is much potential benefit in the complementary application of solid-state NMR spectroscopy and CSP methods. For CSP methods, figuring out the true number of polymorphs that exist and those that have not been found is one of the biggest challenges. Moreover, despite the fact that the CSP methodology explains the range of thermodynamically favored crystal packings very well, even the most robust algorithms still do not consider static or/and dynamic molecular disorder, commonly observed by experimental techniques. These challenges have been discussed, in particular in relation to pharmaceutically important compounds.^40,41^ On the other hand, solid-state NMR spectroscopy is readily applicable to systems exhibiting conformational flexibility and/or different intermolecular interactions.

One of the simplest and most common applications of solid-state NMR is the use of a one-dimensional ^13^C cross-polarization (CP) MAS experiment to determine the number of nonequivalent molecules in the asymmetric unit cell: i.e., the crystallographic *Z′* value.^16^ It is a first stage of the strategy called NMR crystallography^42−54^ where an integral part is the gauge-including projector-augmented wave (GIPAW) method^55−57^ for the calculation of NMR parameters that is a landmark development in theoretical predictions of NMR parameters for solid materials. This approach can be readily applied to provide experimental verification of CSP solutions; specifically, it is indicative of whether an identified likely solution in the CSP strategy corresponds to an experimental form.^12,33,58−60^

**Figure 1:**
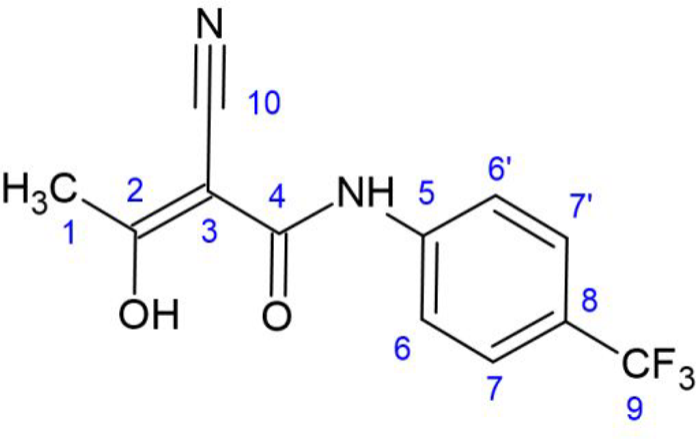
Chemical structure of teriflunomide, including the numbering scheme used here.

This paper shows the synergy of experimental solid-state NMR and single crystal X-ray diffraction methods as well as thermal analysis with DFT calculations and the crystal structure prediction technique in the analysis of a system forming polymorphs, undergoing phase transitions and local dynamic processes. Specifically, teriflunomide (**TFM**) is a moderately sized API (Figure 1) approved for multiple sclerosis treatment in 2012 by the FDA, under the brand name Aubagio.^61,62^ Teriflunomide has also been recently applied as a noninvasive drug administered to the brain that easily bypasses the blood–brain barrier in glioblastoma treatment.^63^

Recently Gunnam et al.^64^ have reported a low-temperature single-crystal X-ray diffraction structure of **TFM** (CSD reference code: 1885431) as well as **TFM** cocrystal structures. In this paper, we identify a polymorphic transformation at −40 °C that has not been reported by Gunnam et al. Our paper applies a complementary multitechnique approach, employing 1D and 2D solid-state MAS NMR techniques, low- and room-temperature X-ray diffraction measurements, and differential scanning calorimetry (DSC) as well as the DFT-based GIPAW calculation of NMR chemical shifts and crystal structure prediction (CSP).

## 2 Results and Discussion

### 2.1 Variable-Temperature Solid-State NMR study of Teriflunomide

As highlighted in the Introduction, the first step in a solid-state NMR analysis of a crystalline organic sample as part of a structure assignment strategy is to answer the following question: what is the number of independent molecules in the asymmetric part of a crystallographic unit, labeled as *Z′*? In the simplest case, there is a straightforward correlation between the number of NMR signals in the isotropic part of spectrum and *Z′*. In the Introduction, we also emphasized that the determination of the exact *Z′* value is not always obvious. In some cases, reported in the literature, deducing whether this value is equal to 1 or more is ambiguous.^4^

In our studies with **TFM**, we began by carefully selecting a representative material. In order to obtain good-quality samples for the structural investigations, several crystallizations of teriflunomide were carried out by employing various organic solvents. The preliminary solid-state NMR studies proved that, despite numerous efforts and optimization of the crystallization conditions, all obtained samples appeared to be of the same polymorphic form. The results presented in this paper are for **TFM** crystallized from dichloromethane; a powder X-ray diffraction (PXRD) pattern is shown in Figure S1.

**Figure 2:**
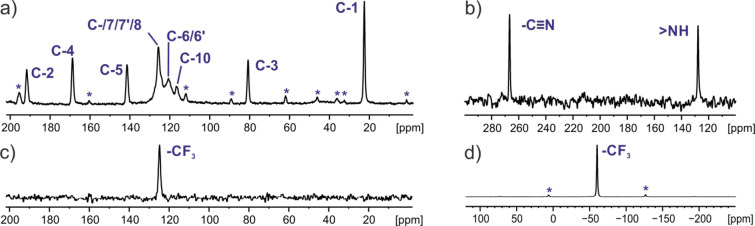
^1^H → ^13^C (a), ^1^H → ^15^N (b), and ^19^F → ^13^C (c) CP MAS and one-pulse ^19^F MAS (d) NMR spectra of **TFM** recorded at spinning rates of 12 (a, b) and 25 kHz (c, d) at 28 °C at 14 T, corresponding to a ^1^H Larmor frequency of 600.1 MHz. For a recycle delay of 45 s, 1024, 8192, 2048, and 16 transients were coadded for (a)–(d), respectively. Asterisks indicate spinning sidebands.

Figure 2a–c shows ^1^H → ^13^C, ^1^H → ^15^N, and ^19^F → ^13^C one-dimensional CP MAS NMR spectra, respectively, and Figure 2d shows a one-pulse ^19^F MAS NMR spectrum recorded at 28 °C. Note that magnetization transfer from fluorine to ^13^C (Figure 2c) was performed because the carbon-13 signal from the −CF_3_ group is missing in classical ^1^H → ^13^C CP MAS NMR spectra. In all spectra, narrow resonance lines confirm that the sample is well ordered and is highly crystalline. When the number of resonance lines in the isotropic part of the spectra are accounted for, it is evident that, for the sample under investigation, the *Z’* value equals 1. Such information is in conflict with X-ray data reported by Gunnam et al.^64^ Indeed, our ^1^H → ^13^C CP MAS NMR spectrum in Figure 2a is the same as the room-temperature ^13^C CP MAS spectrum presented by Gunnam et al. in Figure S3b, but they do not comment on the absence of a doubling of ^13^C resonances.

**Figure 3:**
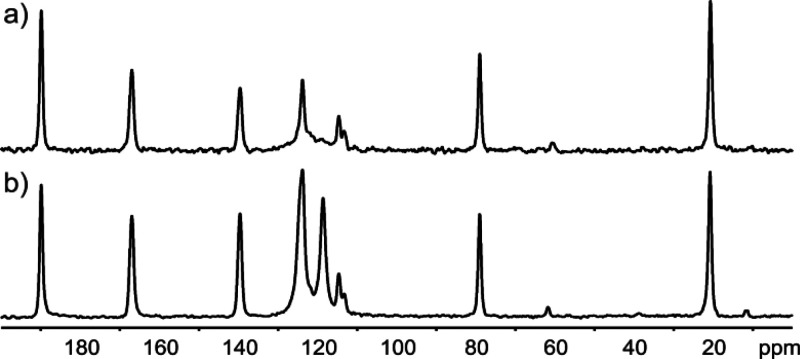
^1^H → ^13^C CP MAS NMR spectra of **TFM** recorded at 40 °C (a) and 20 °C (b) with a spinning rate of 13 kHz and a ^1^H Larmor frequency of 600.1 MHz. A total of 256 transients were coadded for a recycle delay of 60 s.

In a further analysis of the ^1^H → ^13^C CP MAS NMR spectrum, a feature that is ambiguous and surprising is the intensity of signals in the aromatic region. The intensity of the CH carbons (6, 6′, 7, and 7′) is unexpectedly weak, and changing the CP MAS experiment setup, specifically the nutation frequencies and contact time for CP and the relaxation delay, did not change this. However, Figure 3 shows that the signal intensity for these aromatic carbons increases noticeably upon changing the temperature from 40 to 20 °C. This observation is indicative of temperature-dependent phenyl ring dynamics that slow upon a decrease in the temperature (see further discussion below in section 2.4).^65^

**Figure 4:**
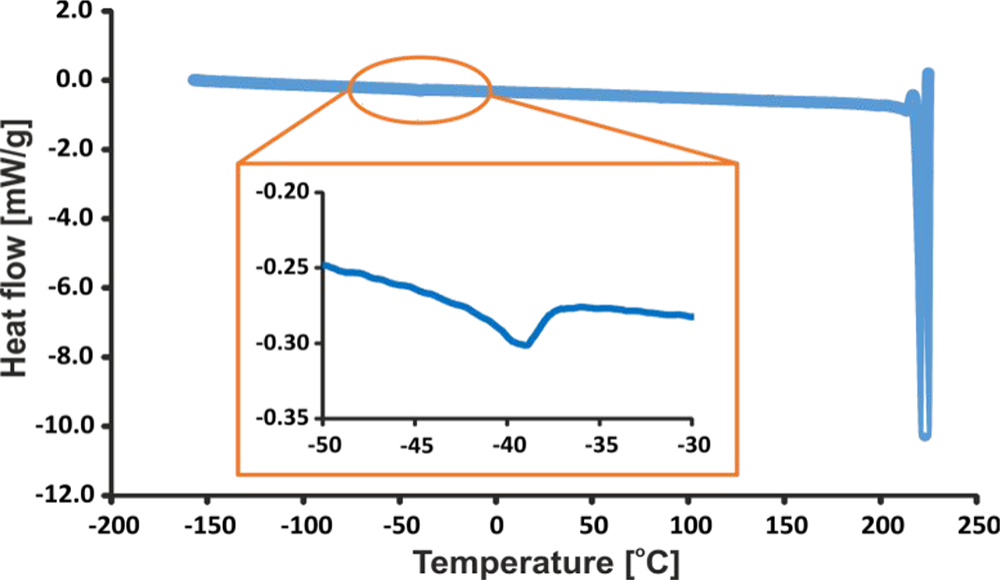
DSC plot for the **TFM** sample with a heating rate of 5 °C min^–1^.

Given the observation of dynamics in the sample, it is instructive to consider Figure 4, which shows a DSC plot for the **TFM** sample. The heating program, from −150 °C up to 250 °C, shows a strong endothermal peak at around 230 °C, which is the melting point temperature. However, a much more interesting feature is evident from a closer look at the region below room temperature. The enlarged inset around −39 °C clearly indicates a very weak endothermal peak. It is worth mentioning that this thermal effect was repeatedly observed in multiple cooling–heating runs. This suggests a reversible transformation that has only a relatively small effect on the organization of the **TFM** crystal structure. Although Gunnam et al.^64^ reported DSC results for **TFM**, the starting point of their DSC curve (in Figure 8) was 30 °C: i.e., too high to observe this thermal transformation.

**Figure 5:**
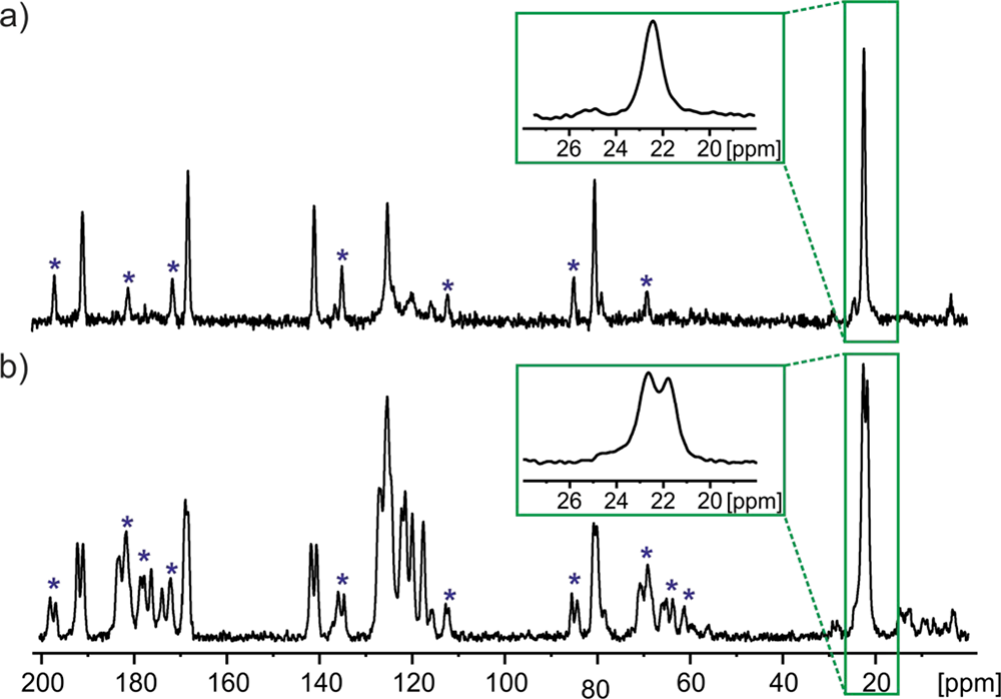
^1^H → ^13^C CP MAS NMR spectra of **TFM** recorded at a spinning rate of 12 kHz with an input gas temperature of (a) 20 °C and (b) −80 °C and a ^1^H Larmor frequency of 850.2 MHz. Totals of 48 and 64 transients were coadded for a recycle delay of 60 s for (a) and (b), respectively. Asterisks indicate spinning sidebands.

With the aim of identifying a correlation between the DSC and solid-state NMR spectroscopy data, we carried out a ^13^C CP MAS NMR experiment at low temperature, below the phase transition point observed by DSC in Figure 4. Specifically, Figure 5 compares ^13^C CP MAS NMR spectra recorded at (a) 20 °C and (b) −80 °C.

From an inspection of the spectra in Figure 5, it is clear that most of the ^13^C resonances appear as two peaks at the lower temperature. This is highlighted for the −CH_3_ group, as shown in the insets in Figure 5. This is unambiguous evidence that the low-temperature **TFM** polymorph has a *Z′* value of 2, in agreement with the low-temperature single-crystal X-ray diffraction structure of **TFM** (CSD reference code 1885431) reported by Gunnam et al. A discussion about the spinning sidebands (denoted by *) seen in Figure 5 can be found in the Supporting Information.

### 2.2 **TFM** X-ray Single-Crystal Structures at 100 and 295 K

In this work, we have obtained single-crystal X-ray structures, for the same crystal kept in the diffractometer, at 100 and 292 K. The crystallographic data for the two polymorphs labeled as **TFM**^**LT**^ and **TFM**^**RT**^ as well as those determined by Gunnam et al.^64^ (CSD code 1885431) are presented in Table 1. Both data sets were indexed in the triclinic system with the *P*1̅ space group, but the *a* cell length is approximately double in the **TFM**^**LT**^ data and the unit cell angles differ (on the frames where a* were visible, reflections were twice as dense). The crystal structures have been deposited in the Cambridge Structural Database under the deposition number 1892916 for **TFM**^**LT**^ and 1969989 for **TFM**^**RT**^. The main difference is the number of molecules in the asymmetric unit cell (the *Z′* values are equal to 1 and 2 for **TFM**^**RT**^ and **TFM**^**LT**^, respectively) in agreement with the solid-state NMR observations.

**Table 1:** Crystal Structure and Refinement Data for **TFM**

	**TFM**^**LT**^	**TFM**(64)	**TFM**^**RT**^
CCDC code	1892916	1885431	1969989
empirical formula	C_12_H_9_F_3_N_2_O_2_	C_12_H_9_F_3_N_2_O_2_	C_12_H_9_F_3_N_2_O_2_
formula wt	270.21	270.21	270.21
temp (K)	100	100	292
cryst syst	triclinic	triclinic	triclinic
space group	*P*1̅	*P*1̅	*P*1̅
*a* (Å)	9.4030(2)	9.3951(5)	4.85221(15)
*b* (Å)	11.5300(2)	11.5161(7)	10.8738(3)
*c* (Å)	11.9572(3)	11.9447(8)	11.7015(3)
α (deg)	95.9600(17)	95.990(3)	102.454(2)
β (deg)	105.8499(19)	105.839(2)	97.821(2)
γ (deg)	110.763(2)	110.753(2)	93.832(2)
*V* (Å^3^)	1136.7	1133.21(12)	594.284
*Z*	4	4	2
*Z′*	2	2	1
density (g cm^–3^)	1.579	1.579	1.510
θ range (deg)	75.391		75.051
index ranges	–11, +11; −14, +14; −14, +14		–5, +6; −13, +13; –14, +14
*N*_ref_ (*I* > 2σ(*I*)	4638 (4442)	5004 (3443)	2413 (2298)
R1 (*I* > 2σ(*I*))	0.0395 (0.0386)	0.0505	0.0448 (0.0438)
wR2 (I > 2σ(*I*))	0.1141 (0.1130)	0.1277	0.1286 (0.1275)

**Figure 6:**
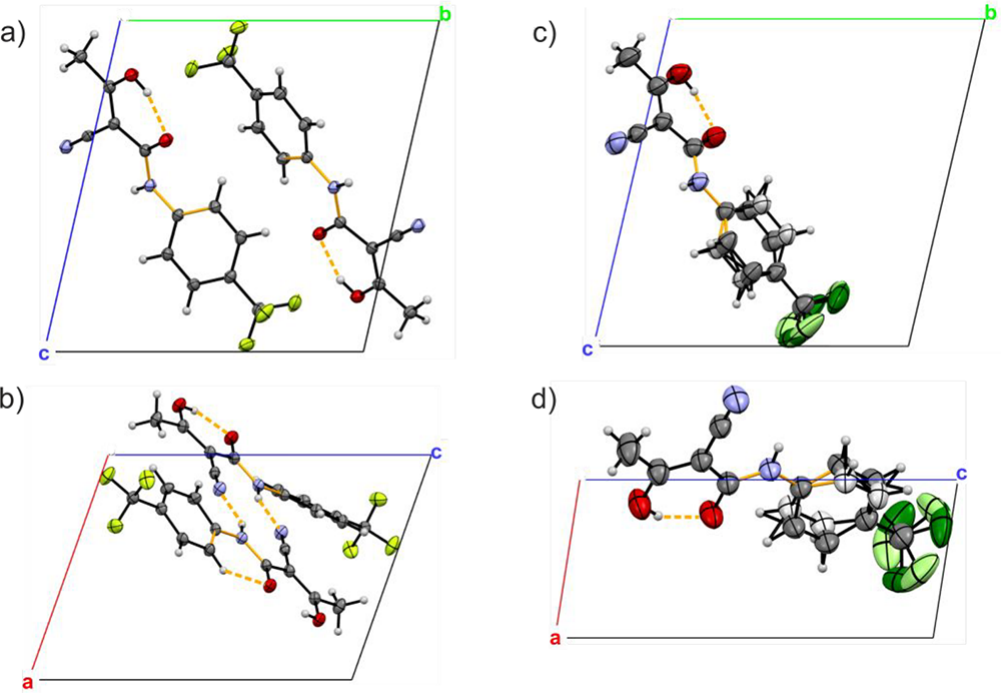
Asymmetric unit cell of (a, b) the **TFM**^**LT**^ and (c, d) the **TFM**^**RT**^ polymorphw displayed along the *a* (a, c) and *b* (b, d) directions. The atoms with a lower occupancy of disordered fragments in the room-temperature structure are marked with a lighter shade of the corresponding color. The C4–NH–C5–C6 torsional angle is highlighted in orange, and the crystallographic axes are shown in red (*a* direction), green (*b* direction), and blue (*c* direction). Intermolecular NH···N and intramolecular OH···O hydrogen bonds (see Table S3) are indicated by orange dashed lines.

Figure 6 shows a comparison of the asymmetric unit cell of the **TFM**^**LT**^ (left, a and b) and **TFM**^**RT**^ (right, c and d) polymorphs. In the **TFM**^**RT**^ structure, disorder of the phenyl ring (corresponding to a rocking movement) and the trifluoromethyl group (rotation around the C–C bond) was observed. Two orientations of disordered parts were refined with occupancy factors summed to unity and each of them close to 0.5, specifically 0.495 for carbon and hydrogen as well as 0.402 for fluorine atoms for the lower occupancy and 0.505 for carbon and hydrogen as well as 0.598 for fluorine atoms for the higher occupancy. Due to different temperature conditions for both measurements, we observed much larger thermal ellipsoids for **TFM**^**RT**^ than for **TFM**^**LT**^. Specifically, the average volumes for the fluorine position was 0.014 and 0.080 Å^3^ for **TFM**^**LT**^ and **TFM**^**RT**^, respectively. The hydrogen atoms connected to carbon atoms and the amide nitrogen were set geometrically and refined as riding with the thermal parameter equal to 1.2 of the thermal vibration of the parent atom. The hydrogen atoms at hydroxyl oxygens were found on the difference Fourier map and refined with geometrical restraints and thermal parameters equal to 1.5 of the thermal vibration of the parent atom. Intermolecular NH···N and intramolecular OH···O hydrogen bonding is indicated by orange dashed lines in Figure 6.

The low-temperature form shows a slight conformational difference of both **TFM** molecules that is mostly located in the values of the C4–NH–C5–C6 torsional angle (4.5 and 31.9°). Since the **TFM**^**RT**^ structure contains one molecule in the asymmetric part of the unit cell (*Z′* = 1), a corresponding comparison can be performed between the less and more highly occupied positions, for which the C4–NH–C5–C6 torsional angle values are −7.8 and 28.6°, respectively. Table 2 presents a comparison of C4–NH–C5–C6 torsional angle values for the experimental diffraction structures as well as those after DFT-D (CASTEP) geometry optimization. It is interesting that both **TFM**^**RT**^ lower and higher occupancy sites (C4–NH–C5–C6 equal to −7.8 or 28.6°) lead to almost the same final structure with a torsional angle of 4.7 or 5.1° after geometry optimization of all atomic positions with the unit cell parameters fixed to those determined by X-ray diffraction. Optimizing all atomic positions and additionally allowing the unit cell parameters to vary again results in very similar C4–NH–C5–C6 torsional angles of 4.2 and 5.7°. Table S1 in the Supporting Information gives the modified unit cell parameters: the changes in unit cell parameters being similar to those observed previously.^43,66^Table S2 states the energy differences for the different DFT-D calculations, with DFT having previously been used to evaluate different crystal structures.^67−69^ Note that for **TFM**^**RT**^, with the less or more highly occupied sites only as starting points, i.e. C4–NH–C5–C6 torsional angle of −7.8 and 28.6° (labeled as A and B), respectively, there remains an energy difference of only 0.8 kJ/mol after optimization of all atomic positions and unit cell parameters.

**Table 2:** C4–NH–C5–C6 Torsional Angle (deg) Values in **TFM** for Single-Crystal X-ray Diffraction Structures and Those Obtained after DFT-D (CASTEP) Geometry Optimization

	C4–NH–C5–C6 torsional angle		
structure	X-ray data	DFT-D (opt all)^a^	DFT-D (opt all + cell)^b^
**TFM**^**LT**^ (molecule A)	4.5	3.4	4.2
**TFM**^**LT**^ (molecule B)	31.9	29.1	29.3
**TFM**^**RT**^ (lower A occupancy sites)^c^	–7.8	5.1	4.2
**TFM**^**RT**^ (higher B occupancy sites)^c^	28.6	4.7	5.7

a DFT-D geometry optimization of all atomic positions with the unit cell parameters fixed to those determined by X-ray diffraction.

b DFT-D geometry optimization of all atomic positions and allowing the unit cell parameters to vary (see Table S1 in the Supporting Information)

c The DFT calculations for the lower A and higher B occupancy sites of **TFM**^**RT**^ were performed for two separate systems where the starting unit cell contains the experimental A or B sites only.

**Figure 7:**
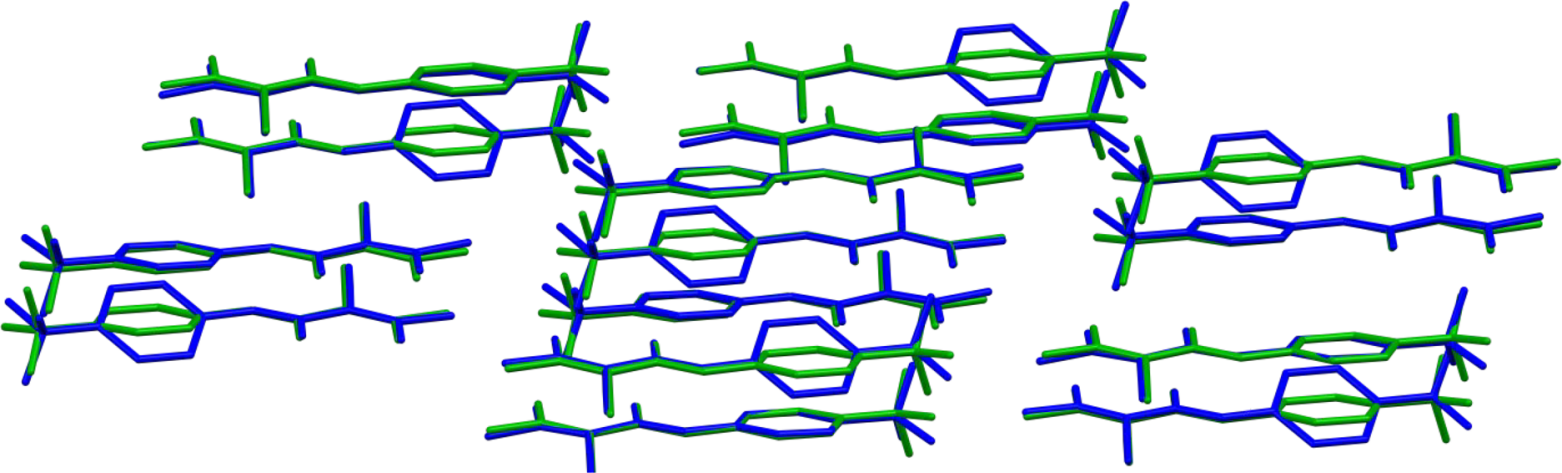
Superposition of clusters within the COMPACK algorithm^70^ for **TFM**^**LT**^ (blue) and **TFM**^**RT**^ (green) containing 15 TFM molecules (only non-hydrogen atoms are shown). Note that the COMPACK algorithm comparison procedure considers only the most highly occupied sites.

It is to be stressed that the packings of both crystal structures are highly similar to each other. We used the COMPACK algorithm, which is well suited to comparing two crystal structures and determining their metric of similarity.^70^ The idea is based on the construction and superposition of clusters for comparing crystal forms and the calculation of the difference (root mean square deviation, or RMSD value) among equivalent atomic positions. Figure 7 shows the COMPACK superposition of the clusters **TFM**^**LT**^ and **TFM**^**RT**^ containing 15 TFM molecules. Note that the COMPACK algorithm comparison procedure presented here considers only the most highly occupied sites.

The calculated RMSD_15_ is equal to 0.396 Å. The value is not large, and a closer inspection of the clusters shows that the main differences concern the orientation of the aromatic ring as well as the fluorine atoms in the −CF_3_ group. It also cannot be excluded that there is an effect whereby the orientation of the phenyl ring is correlated with the orientation of the −CF_3_ group.

Since the fluorine atoms in the **TFM**^**RT**^ structure are affected by positional disorder, it seems to be justified not to consider fluorine atoms belonging to the −CF_3_ group during the comparison of both polymorphs. In such a case, the appropriate RMSD_15_ value (without the disordered −CF_3_ group and aromatic positions) drops to 0.149 Å. The resulting RMSD_15_ proved that the same long-range order for both **TFM**^**LT**^ and **TFM**^**RT**^ structures is preserved. Moreover, on consideration of the intermolecular NH···N and intramolecular OH···O hydrogen bonding, Table S3 in the Supporting Information shows that, after DFT-D geometry optimization of all atomic positions and the unit cell parameters being allowed to vary, the N···N and H···N distances, and the NHN angle and the O···O and H···O distances, and the OHO angle only vary between 2.94 and 2.99 Å, 1.93 and 1.98 Å, and 164 and 166° and between 2.44 and 2.46 Å, 1.45 and 1.47 Å, 154 and 155°, respectively.

We further note that, in the paper by Gunnam et al.,^64^ it is stated on page 5411 that there is a match of the PXRD experimental line pattern and the calculated line profile from the X-ray crystal structure, even though we have discovered the anomaly in the *Z′* value. This observation can be understood, since we observed a great similarity of both simulated PXRD patterns based on **TFM**^**RT**^ and **TFM**^**LT**^ single-crystal X-ray diffraction structure solutions (see Figure S2): noticeable deviation is only observed for 2θ values above 18°.

### 2.3 NMR Crystallography of the **TFM** Room-Temperature Polymorph

**Figure 8:**
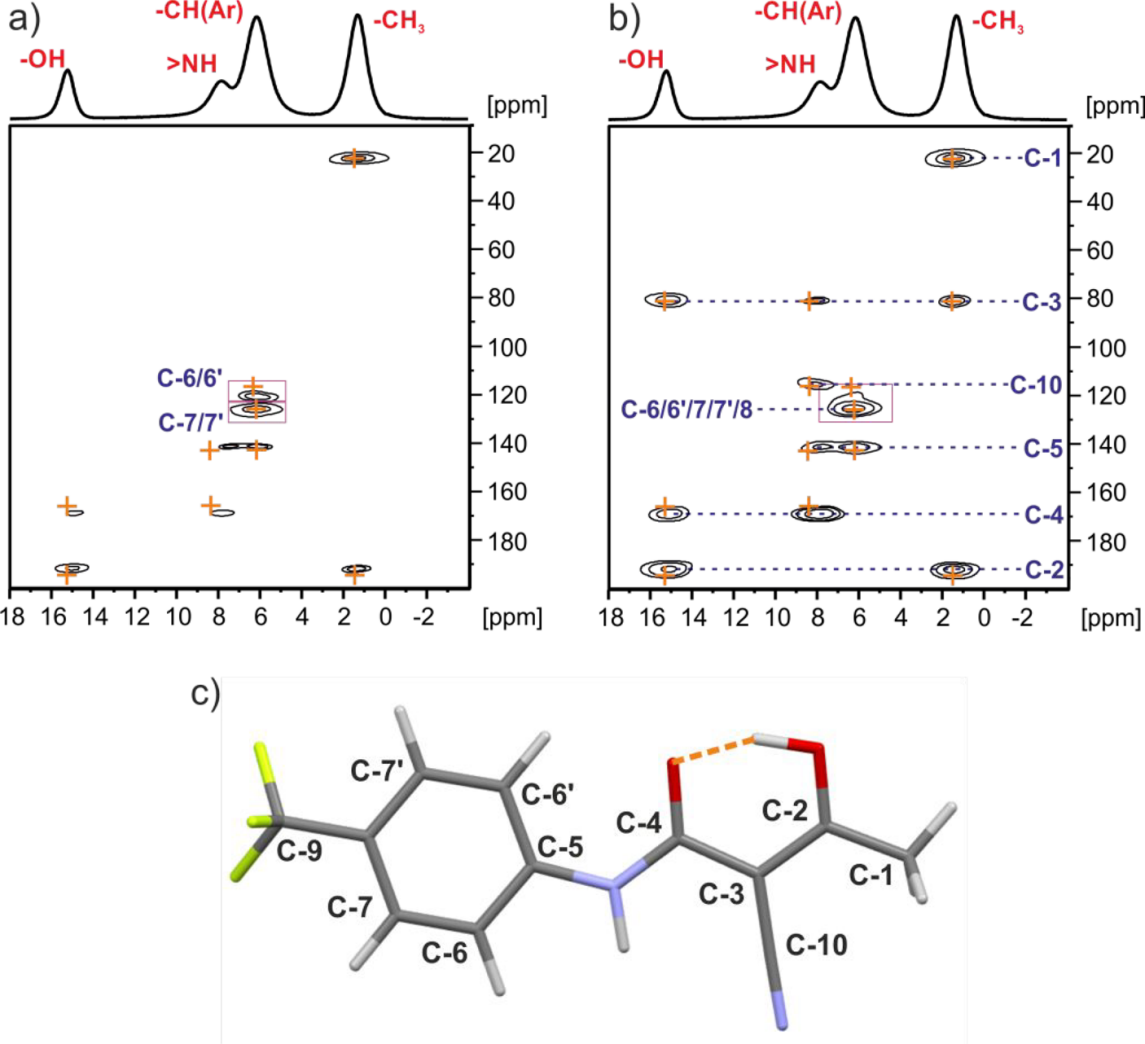
^13^C–^1^H invHETCOR MAS NMR spectra of **TFM**^**RT**^ recorded at 20 °C with a spinning rate of 62.5 kHz at a ^1^H Larmor frequency of 600.1 MHz with a second (^13^C → ^1^H) CP contact time of (a) 150 μs and (b) 1000 μs. A one-pulse ^1^H MAS spectrum is shown at the top. The orange crosses represent GIPAW calculated NMR correlations for C···H distances up to (a) 2.2 and (b) 3.0 Å. The base contour is at 10% of the maximum peak intensity. (c) Pictographic representation of the OH···H intramolecular hydrogen bond (orange dotted line) in the **TFM**^**RT**^ structure.

The results presented in sections 2.1 and 2.2 show that **TFM** exhibits temperature-dependent polymorphism. This section extends the solid-state MAS NMR characterization of the room-temperature polymorph (*Z′* = 1), specifically also presenting ^1^H NMR spectra recorded under fast MAS. In particular, this enables the recording of 2D heteronuclear experiments with indirect inverse (inv) observation via ^1^H such as a ^13^C–^1^H invHETCOR MAS NMR experiment that utilizes ^1^H → ^13^C and ^13^C → ^1^H CP before and after *t*_1_ evolution, respectively. Figure 8 shows ^13^C–^1^H invHETCOR MAS spectra acquired with a spinning rate, ν_R_ = 62.5 kHz, with (a) short (150 μs) and (b) long (1 ms) ^13^C → ^1^H CP contact times such that only cross peaks corresponding to short C···H distances, mostly direct C–H bonds, are observed in Figure 8a, while cross peaks due to longer range C···H proximities are seen in Figure 8b.

On consideration of the ^13^C–^1^H invHETCOR MAS NMR spectrum in Figure 8a, as well as the peaks for one-bond C–H connectivities, C6/6′, C-7/7′, and the CH_3_ methyl group, note the strong intensity of correlation peaks between the OH proton and quaternary C2 and C4 carbons that is consistent with an intramolecular hydrogen bonding between the OH group and the carbonyl group, as shown in Figure 8c (see also Figure 6). In Figure 8b, additional strong cross peaks between C3 and the methyl and OH protons are observed as well as between the NH protons and the C3, C4, C5, and C10 carbons, as is consistent with the molecular conformation shown in Figure 8c.

Verification of structures determined by X-ray diffraction (or also CSP) can be achieved by comparing experimental solid-state NMR chemical shifts with GIPAW-calculated chemical shifts.^34,42,44,45,66,71^ The orange crosses in Figure 8 correspond to the NMR correlations based on GIPAW calculations for the **TFM**^**RT**^ crystal structure (after DFT geometry optimization). Excellent agreement is observed between experimental and GIPAW-calculated chemical shifts, as reflected by the small root-mean-squared error (RMSE) values of 0.14 and 2.2 ppm for ^1^H and ^13^C, respectively (Table 3).^34,42,44,45,66,71^ (Note that the GIPAW-calculated shieldings for **TFM**^**LT**^ and **TFM**^**RT**^ are given in Table S4.) In this context, for organic molecules, Emsley and co-workers have established a threshold root-mean-square error (RMSE) of 0.3 ppm for ^1^H,^72,73^ while in further work, they establish RMSEs compared to a machine-learning method of 0.5 ppm for ^1^H and 4.3 ppm for ^13^C.^74^ The RMSE calculation for ^13^C does not take into account C-9 that is bonded to fluorine atoms and for which there is a very large discrepancy between experimental and GIPAW-calculated values. In this context, Dybowski and co-workers have identified that such discrepancies arise in DFT calculations of ^19^F magnetic shielding.^75^

**Table 3:** Experimental and GIPAW^a^-Calculated ^13^C and ^1^H NMR Chemical Shifts, δ (in ppm), for **TFM**^**RT**^

	δ(^1^H)		δ(^13^C)	
structure (position)	exptl	GIPAW	exptl	GIPAW
1	1.5	1.7^b^	22.3	23.6
2			191.5	194.4
3			80.6	80.5
4			168.6	166.2
5			141.3	141.7
6	6.1	6.1^b^	120.5	118.5^b^
7	6.4	6.2^b^	125.6	126.3^b^
8			125.6	122.5
9			124.8	138.3
10			116.5	120.2
–NH–	8.1	8.3		
–OH	15.2	15.2		

a The GIPAW-calculated chemical shifts were determined from the GIPAW-calculated shieldings (see Table S4) using the following equations: δ(^1^H) = −0.910σ(^1^H) + 27.0 (ppm) and δ(^13^C) = −0.969σ(^13^C) + 167.1 (ppm). Values were obtained after DFT-D geometry optimization, with the unit cell allowed to vary.

b The average of the distinct ^1^H and ^13^C chemical shifts is presented.

**Figure 9:**
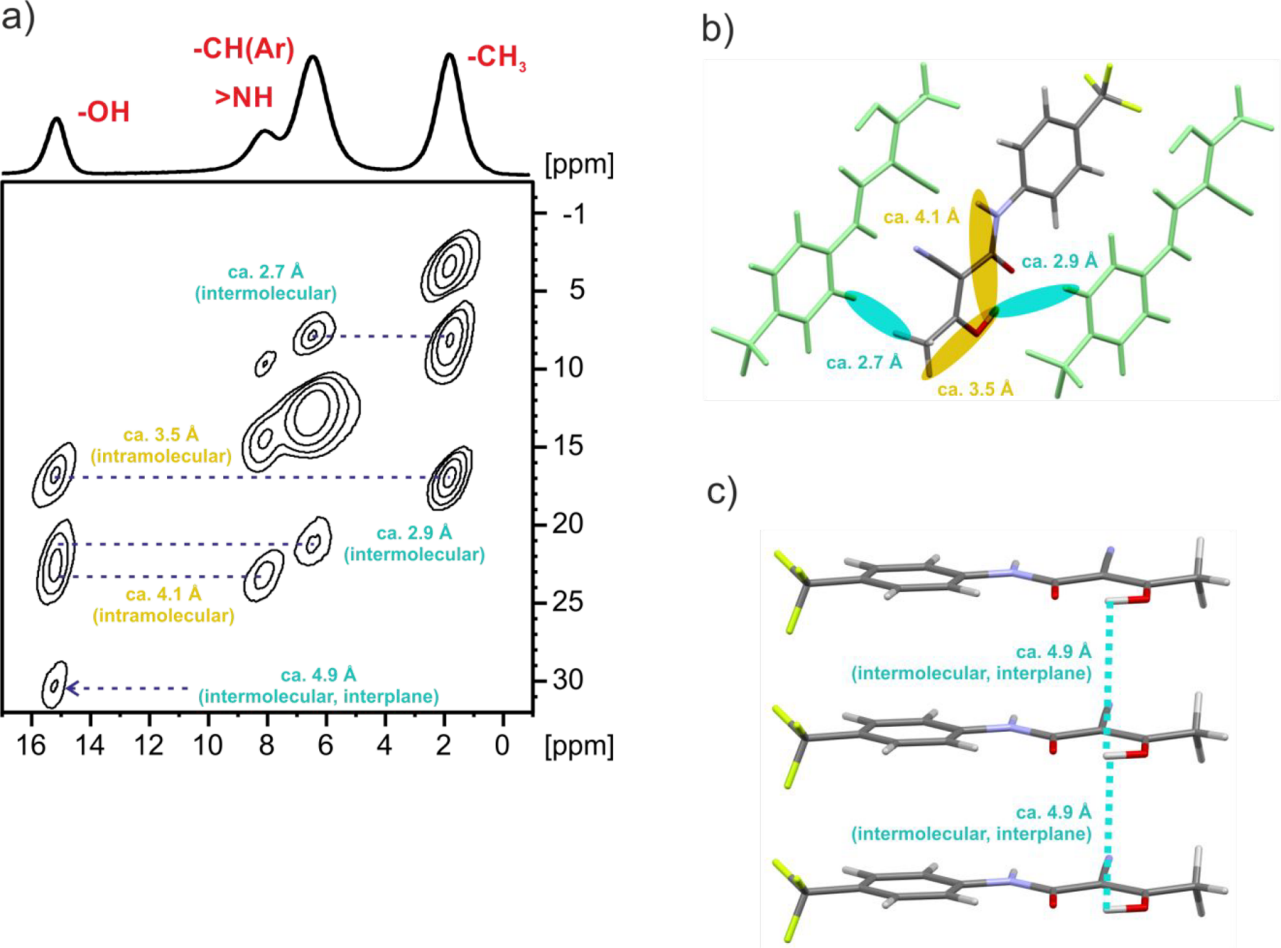
(a) ^1^H–^1^H DQ-SQ MAS NMR spectrum of **TFM**^**RT**^ recorded with one rotor period of BaBa recoupling at ambient temperature and 20 °C with a spinning rate of 62.5 kHz and a ^1^H Larmor frequency of 600.1 MHz. Blue dotted lines and descriptions indicate observed inter- and intramolecular H–H proximities. The base contour is at 5% of the maximum peak intensity. A one-pulse ^1^H MAS NMR spectrum is shown at the top. (b, c) Representations of the observed H–H proximities for the OH and NH in the **TFM**^**RT**^ crystal structure. The H–H distances in the **TFM**^**RT**^ crystal structure after DFT-D geometry optimization are as follows: CH_3_···HC(Ar), 2.71 Å; CH_3_···HO, 3.49 Å; (Ar)CH···HO, 2.92 Å; NH···HO, 4.16 Å; OH···HO, 4.87 Å.

Further structural information is provided by a 2D homonuclear ^1^H double-quantum (DQ) single-quantum (SQ) MAS NMR correlation experiment with back to back (BaBa) recoupling sequence that establishes proximities between specific protons, as shown in Figure 9.^76−78^ Of note is the auto OH DQ peak that is due to an intermolecular OH···HO correlation corresponding to the proximity of **TFM** molecules in different planes (see Figure 9c). The expected relative intensities of correlation peaks H(i)···H(j) and H(i)···H(k) can be estimated from the proton–proton proximities using the ∼*r*_ij_^6^/*r*_ik_^6^ formula, where *r*_ij_ and *r*_ik_ are distances between the H(i)···H(j) and H(i)···H(k) coupled protons, respectively.^76^ For **TFM**^**RT**^, the OH···HO inteplanar distance is 4.87 Å; thus, it is expected that the peak intensity will be only 2.92^6^/4.87^6^ = 0.05 of the CH(aromatic)···HO peak intensity (this is the closest proton to the OH). This is doubled, since there are two such interplanar OH proximities; thus, a 10% relative intensity is consistent with that seen experimentally in Figure 9a.

### 2.4 Local Molecular Dynamics in the Crystal Lattice of **TFM**^**RT**^

**Figure 10:**
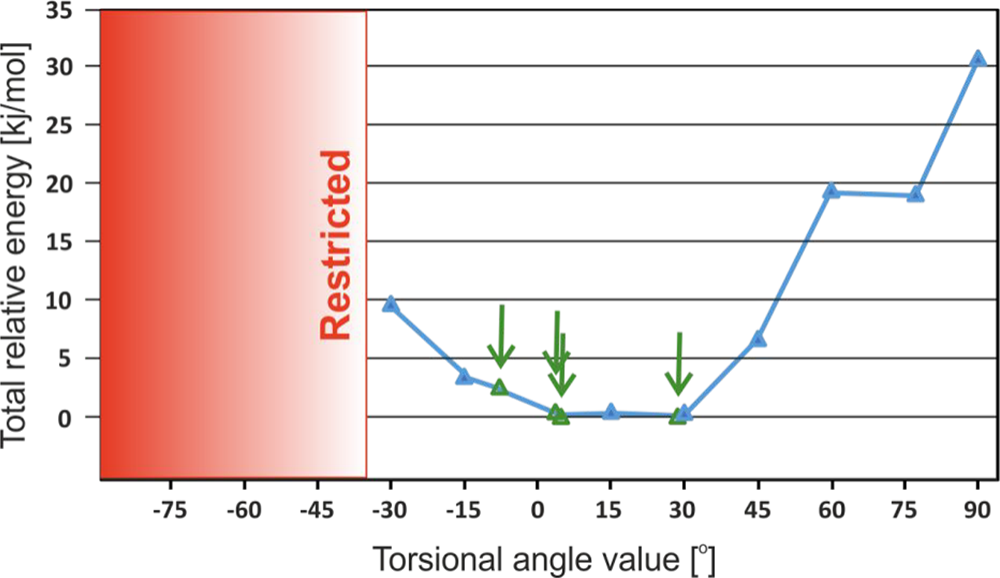
Energy around the equilibrium position corresponding to the change in DFT-calculated energy for different C4–NH–C5–C6 torsional angles in the full periodic **TFM**^**RT**^ structure. The angles observed in the **TFM**^**RT**^ crystal structure (namely, −7.8 and 28.6° and the 4.7 and 5.1° values after DFT-D geometry optimization) are indicated by green arrows and green triangles. Energy values of 109.9, 196.4, and 115.9 kJ/mol were calculated for torsional angles of −75, −60, and −45°, respectively; thus, the red area is labeled as restricted. Blue lines linking the symbols are included as a guide to the eye.

As noted above, the variable-temperature ^13^C CP MAS spectra displayed in Figure 3 indicate phenyl ring dynamics in the room-temperature form. Possessing the single-crystal X-ray solution for **TFM**^**RT**^ allowed us to investigate the possibility of a dynamic process, by means of theoretical methods. Specifically, following an approach used elsewhere,^79−82^ we investigate by DFT calculations the effect of changing the C4–NH–C5–C6 torsional angle value which causes aromatic ring rotation around the 1–4 axis. In our procedure, due to the plane symmetry of the aromatic moiety, the torsional angle value was incremented from −90° to +90° (equivalent of −90°) with a 15° interval (omitting the value 0°). Additionally, we added in (green triangles and arrows) values from Table 2 for the **TFM**^**RT**^ structure (namely, −7.8 and 28.6° from the diffraction structure as well as 5.1 and 4.7° after DFT-D geometry optimization). Figure 10 shows a plot of the energetics around the equilibrium position upon changing the C4–NH–C5–C6 torsional angle. The red area represents the region where the total relative energy was higher than 35 kJ/mol. Both crystallographically observed torsional angle values are found in the energetic minima; however, the 28.6° torsion angle is more preferred by 2.6 kJ/mol to the −7.8° torsion angle. Note that the DFT-D optimization of the full crystal structure gives the lowest energy point at a torsion angle of 5.1° (see Table 2 and Table S2). The energy change in the wider range between −30 and 45° of the C4–NH–C5–C6 value is lower than 10 kJ/mol, with this value corresponding to an upper limit on typically experimentally accessible rotational barriers, as assessed by literature studies that report on discrete DFT calculations.^79−82^ Note that such DFT calculations only probe the thermodynamics (i.e., equivalent to 0 K) and temperature-dependent kinetic effects are not considered.

In addition to the DFT calculations that explored the energetic barrier corresponding to changing the C4–NH–C5–C6 torsional angle, as presented in Figure 10, this section presents 2D ^1^H–^13^C PISEMA MAS NMR spectra by which the changes in the ^13^C–^1^H dipolar couplings due to motion of the phenyl rings are quantitatively determined.

Solid-state NMR spectroscopy offers a rich palette of methodological approaches that allows the study of molecular motion on different time scales. Usually, the choice of technique is correlated with the nature of dynamic processes. In our case, we find 2D PISEMA MAS experiments^83^ to be a useful diagnostic tool. This is a well-established solid-state NMR method to measure ^13^C–^1^H dipolar couplings and probe dynamic processes on the microsecond time scale.^84,85^ The splitting between the singularities in the *F*_1_ dimension of 2D PISEMA MAS spectra reflects the dipolar coupling between the specific carbon and closely located protons. According to the equation *D* = −(μ_0_ℏ/8π^2^)(γ_*i*_γ_*j*_*)/r*_*ij*_^3^, the dipolar coupling constant for the rigid limit for a ^13^C–^1^H distance equal to 1.09 Å is 23.3 kHz. The experimentally measured splitting values are smaller than the calculated coupling values because the observed splitting is reduced by a scaling factor.^86^ For the PISEMA MAS NMR experiment, the exact Hartmann–Hahn matching condition yields a scaling factor of 0.577 (cos 54.7°), and the expected splitting value is ca. 13.4 kHz (23.3 kHz × 0.577).^87^ Motional processes can be quantitatively probed by measuring the reduction in the dipolar coupling in comparison to the rigid limit.^88−90^

**Figure 11:**
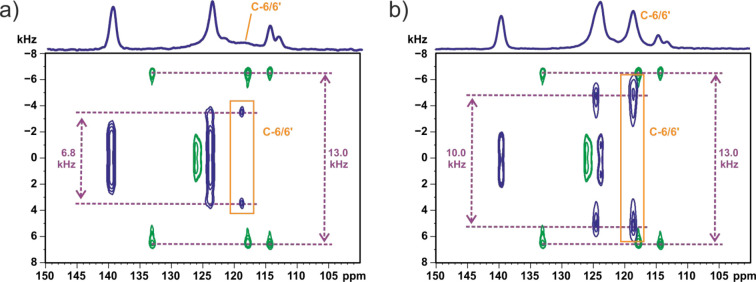
Aromatic region of 2D ^1^H–^13^C PISEMA MAS NMR spectra recorded at 40 °C (a) and 20 °C (b) with a spinning rate of 13 kHz and a ^1^H Larmor frequency of 600.1 MHz for **TFM** (blue) compared to spectra for l-tyrosine hydrochloride (green). ^1^H–^13^C CP MAS spectra recorded with a contact time of 2 ms are shown at the top. The orange rectangles distinguish the relevant C-6/6′ signals of **TFM**.

Figure 11a,b presents 2D ^1^H–^13^C PISEMA MAS NMR spectra for **TFM**^**RT**^ carried out at 40 and 20 °C, respectively. For comparison, spectra obtained for l-tyrosine hydrochloride are superimposed, for which previous studies^91,92^ have shown that the order parameter for the rigid aromatic moiety at these temperatures is close to unity: in Figure 11, the splitting for l-tyrosine hydrochloride is observed to be 13.0 kHz. The observed splittings for the well-separated C-6/6′ aromatic resonances of **TFM**^**RT**^ are 6.8 kHz at 40 °C (Figure 11a), increasing to 10.0 kHz as the sample was cooled to 20 °C (Figure 11b): i.e., corresponding to a scaling compared to the rigid limit of 6.8/13.4 = 51% at 40 °C and 10.0/13.4 = 75% at 20 °C. A simple ring flip corresponds to a scaling by ∼60%.^88,90^ As such, the scaling by 75% at 20 °C can be interpreted as a low-amplitude wobbling of the aromatic ring that is consistent with the broad energy minimum in Figure 10, while the scaling by 51% at 40 °C corresponds to a phenyl flip motion, where there is sufficient energy to overcome the energy barrier in Figure 10. It is interesting to compare the observed values with those in two previous studies by Pawlak et al., specifically, for molecular rotors containing 1,4-diethynylphenylene connected by alkynylene moieties in a steroidal framework^93^ as well as Tyr-Ala-Phe tripeptides with varying alanine residue stereochemistry (L and D).^94^ The scalings for dynamic CH aromatic resonances exhibiting ring flips were in the range 31–51% for **1** and **2b** in ref (94) and **2A** in ref (93), while a scaling of 70% corresponding to small-amplitude wobbling was also observed for **2B** in ref (93). Scalings in the range 89–97% were observed for **2a** in ref (94) as well as for **1A**, **1B**, and **1I** in ref (93).

### 2.5 CSP Validation of Experimentally Obtained Structures

On comparison of the crystal lattice energy computed by means of the DFT-D method, the energy differences between the fully optimized **TFM**^**LT**^ and **TFM**^**RT**^ structures are as much as 2.4 kJ/mol and as small as 0.75 kJ/mol, considering the difference between **TFM**^**LT**^ and **TFM**^**RT**^ (high-occupancy sites in the crystal structure) after geometry optimization of all atomic positions and unit cell parameters (see Table S2). Discerning structures separated by 2.4 kJ/mol or less is challenging for a CSP strategy and can be considered as a test of method sensitivity and reliability. Another challenge is the high similarity of packing of both crystal structures that was stressed in section 2.2 (see Figure 7). The RMSD values as provided by the COMPACK algorithm for the atomic positions are valuable because they determine the limit when the structure obtained from the CSP step can be accepted as a measure of the equivalence to the specific experimental polymorph.^3^

After an examination of both **TFM** crystal structures, the CSP methodology was applied. The procedure included a series of computations using CrystalPredictor as well as CrystalOptimizer algorithms. Further technical details regarding the CSP search are stated in Experimental and Computational Procedures. Extensive calculations with *Z′* = 1 and *Z′* = 2 among the 20 most common space groups (according to the CSD database)^95^ were performed.

**Figure 12:**
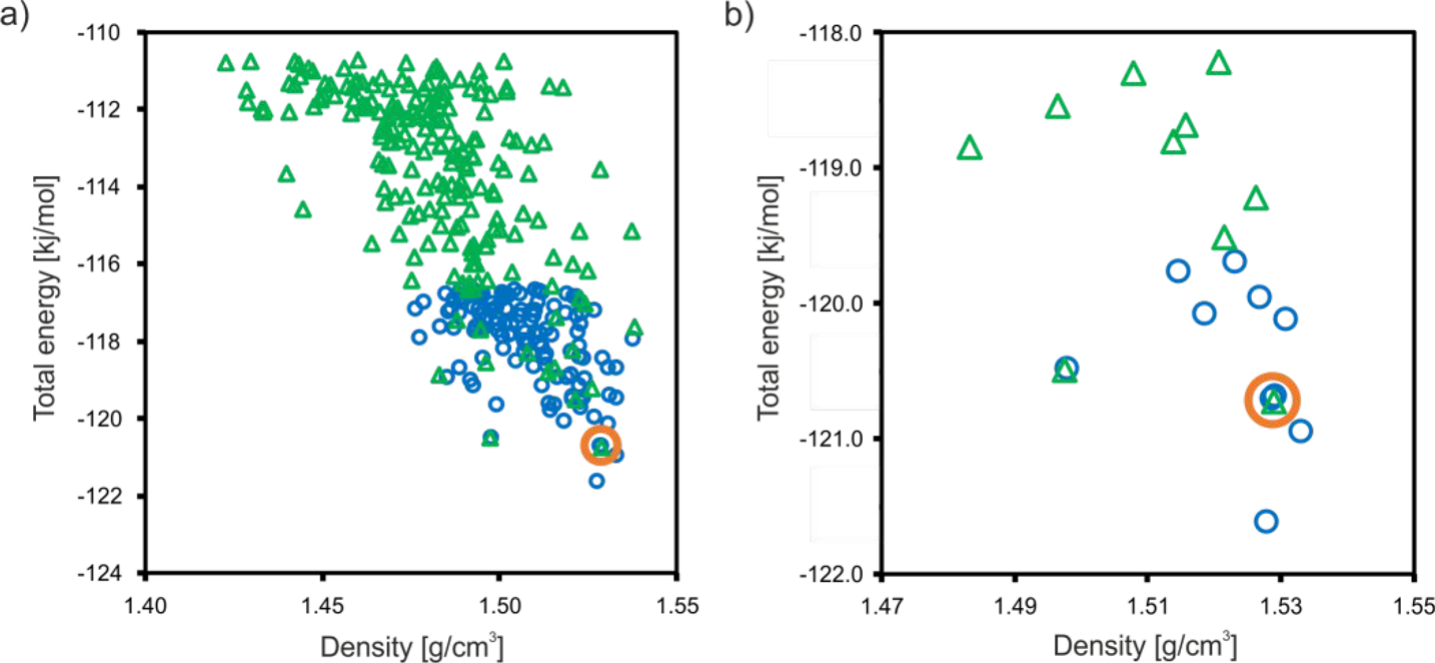
CSP plots for *Z′* = 1 (green triangles) and *Z′* = 2 (blue circles) searches for **TFM** for (a) the 10 kJ/mol energy window and (b) an enlarged region corresponding to the 10 lowest energy structures. The orange circles indicate the CSP “parent” structures best matched to the experimental forms after a subsequent CSP-CASTEP step (see Figure 13 and Tables S5 and S6).

Figure 12 presents CSP plots of lattice energy versus density for the lowest energy structures within a 10 kJ/mol energy window (see also Tables S5 and S6), noting that a recent analysis of over 1000 experimentally determined crystal structures, including over 500 polymorphs of organic molecules, concluded that, in 95% of the cases, the difference in energy between experimentally observed polymorphs is less than 7.2 kJ/mol.^96^ Most of the ca. 200 (*Z′* = 1) and ca. 150 (*Z′* = 2) crystal structures generated within 10 kJ/mol of the global minimum have the same lattice symmetry as the experimental form (*P*1̅). The *Z′* = 2 search dominates the lower energy and higher density region, which shows that this type of crystal is preferred as long as we only consider thermodynamics: i.e., corresponding to 0 K. Moreover, this is consistent with the experimental observation of the conversion of **TFM**^**RT**^ into the more stable low-temperature form (**TFM**^**LT**^) when the temperature is reduced. All of the obtained structures were compared to the experimental structures by using the COMPACK algorithm (as introduced above; see Figure 7). There was only one structure (no. 1) from the *Z′* = 1 search that we can consider as matching to the **TFM**^**RT**^ experimental form on the basis of the RMSD_15_ criteria described above. Unfortunately, none of the results for the *Z′* = 2 search were more similar to the **TFM**^**LT**^ than for the similarity obtained between **TFM**^**LT**^ and **TFM**^**RT**^ forms. The difficulties in this matter are probably because of the fact that both forms are extremely similar to each other, which requires more computationally demanding methods to distinguish them.

**Figure 13:**
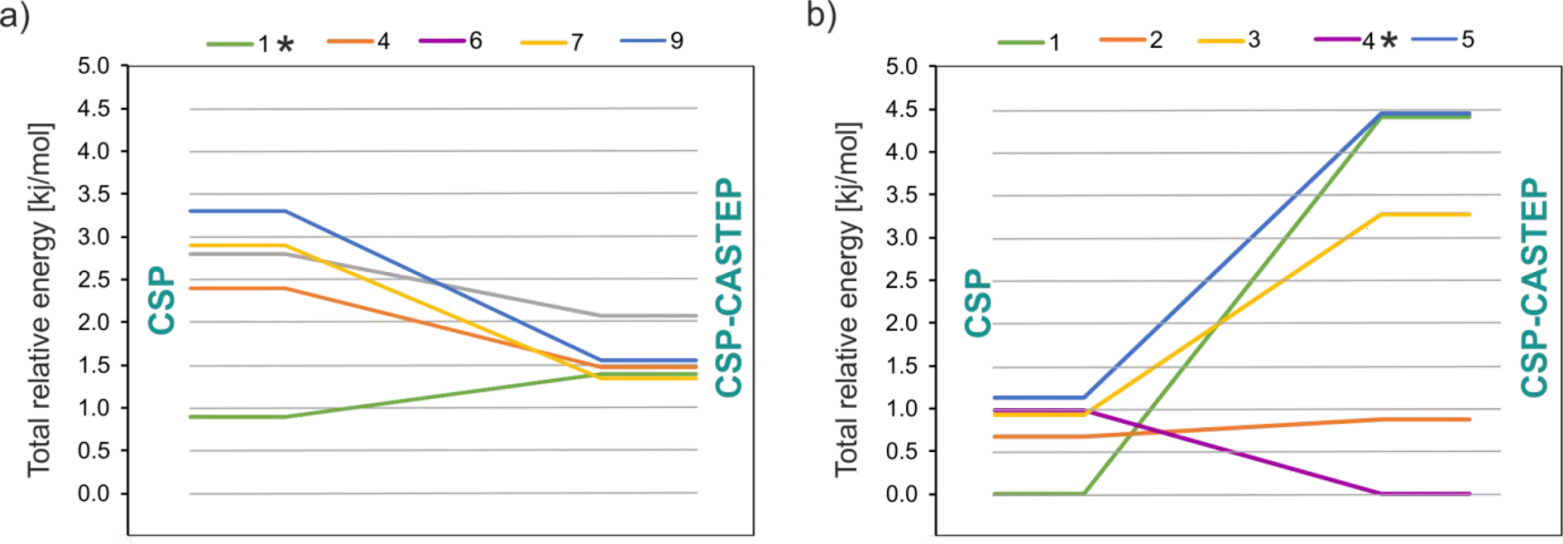
Reordering with respect to energy upon DFT-D (CASTEP) geometry optimization for the (a) *Z′* = 1 and (b) *Z′* = 2 CSP searches for **TFM**. The asterisk indicates the CSP "parent" structure best matched to the experimental form in each case. Both graphs have a consistent zero-point energy level.

As a final evaluation of the CSP-generated structures, DFT-D calculations (implemented in the CASTEP program) were applied—this is referred to here as CSP-CASTEP. The calculations were focused on the optimization of the 10 lowest energy structures obtained from each CSP step (Figure 12b). All atomic coordinates as well as unit cell parameters were allowed to vary. The change in energetic rankings for the five lowest energy CSP-CASTEP structures including the “parent” structures are shown in Figure 13 (see also Tables S5 and S6). The structures obtained after the CSP-CASTEP step were compared to the experimental structures. It was found that the lowest energy structure obtained after CSP-CASTEP for the *Z′* = 2 search and the second lowest energy structure for the *Z′* = 1 search (CSP no. 1) match very well the experimental forms: after DFT-D (CASTEP) geometry optimization, the RMSD_15_ values are only 0.276 and 0.222 Å for the **TFM**^**RT**^ and **TFM**^**LT**^ polymorphs, respectively. These CSP structures are circled in Figure 12b. It is evident that both CSP-CASTEP “parent” structures almost overlay each other in the CSP plot. On the other hand, this is not surprising if we consider the close similarity of the polymorphs **TFM**^**LT**^ and **TFM**^**RT**^ diffraction structures after DFT-D geometry optimization (see also the above discussion of Table S2).

Additionally, COMPACK results and energy differences for the 10 lowest energy structures after DFT-D geometry optimization for *Z′* = 1 and *Z′* = 2 are given in Tables S5 and S6, respectively. It is clearly seen that a significant reordering of energetic preferences has occurred. These changes are much larger for the *Z′* = 2 search than for the *Z′* = 1 search. For the *Z′* = 1 search, the structure that is best matched to experiment is finally placed as the second in the CSP-CASTEP energetic ranking. However, the difference is only 0.03 kJ/mol above the most preferred structure, which is well within the accuracy limit of the computational methods.^97−99^ It is also not meaningless that the *Z′* = 1 search was burdened by the fact that the molecular disorder observed at room temperature is not modeled by the applied computational methods. The *Z′* = 2 search is much less ambiguous and indicates structure no. 4 as the most energetically stable (considering only thermodynamics: i.e., at 0 K corresponding to the zero-point vibration energy level), which is best matched to the experimental **TFM**^**LT**^ structure. Since there are a couple of structures within the threshold of 2 kJ/mol from the lowest energy structure, which is a good estimation of the accuracy of the CSP-CASTEP method,^97−99^ we cannot eliminate the possibility of formation of other unknown yet experimental polymorphs. It is, however, unlikely if we consider the significant experimental efforts devoted to experimental polymorph screening in this study.

## 3 Conclusions

In this work for the first time we present an X-ray diffraction structure and characterization by MAS NMR of a new, room-temperature polymorph of **TFM** (labeled as **TFM**^**RT**^). We observe that the low-temperature TFM polymorph **(TFM**^**LT**^) recently reported by Gunnam et al. undergoes a thermal transition at −40 °C to the **TFM**^**RT**^ polymorph. The phase transition is clearly visible on the DSC plot and is reversible. A crystal mounted on the goniometer head can be cooled and warmed without cracking, producing diffraction patterns characteristic for **TFM**^**LT**^ and **TFM**^**RT**^ lattices, respectively. This reversible process (**TFM**^**LT**^ ↔ **TFM**^**RT**^) occurs with a change in *Z′* value (from 2 to 1) while the crystallographic system is preserved (triclinic). The two forms have different lattice parameters and show different reflection patterns. Every second *h* reflection corresponding to lattice direction *a* in **TFM**^**LT**^ disappeared on **TFM**^**RT**^ diffraction images. We emphasize that both structures have been determined on the basis of diffraction experiments conducted on the same species of crystal.

Our consideration of differences between **TFM**^**LT**^ and **TFM**^**RT**^ focuses on changes in the orientation of the aromatic ring associated with the C4–NH–C5–C6 torsional angle. In **TFM**^**LT**^, there are two torsion angles of 4.5 and 31.9° for the two *Z′* = 2 molecules, while in the room-temperature structure, there is disorder that is modeled with ∼50% occupancy between torsion angles of −7.8 and 28.6°. These observations are consistent with the broad energy minimum observed by DFT calculations for changes in the C4–NH–C5–C6 torsional angle. PISEMA solid-state NMR experiments show a reduction in the C–H dipolar coupling in comparison to the static limit for the aromatic CH moieties of 75% and 51% at 20 and 40 °C, respectively, that is indicative of ring flips at the higher temperature. The difference in crystal lattice energy computed by means of DFT-D between **TFM**^**LT**^ and **TFM**^**RT**^ is up to 2.4 kJ/mol. Such a small difference supports the experimentally observed reversible transformation between both forms.

The **TFM**^**RT**^ polymorph also exhibits molecular disorder of the CF_3_ group, and the disorder in the two parts of the molecule hence may be correlated. The transformation from **TFM**^**LT**^ and **TFM**^**RT**^ appears to be driven by an entropic preference similar to that in a previous study.^100^ It is curious to compare the case here for **TFM**, where disorder in **TFM**^**RT**^ is associated with a change in comparison to **TFM**^**LT**^ in the *Z′* value from 2 to 1, with that presented by Szell et al., where disorder leads to a change in *Z′* value from 1 to 2.^101^

It could be thought that **TFM**^**LT**^ and **TFM**^**RT**^ are not distinct polymorphs but rather the same form with disorder above −40 °C. However, there are clear differences in the calculated PXRD patterns that cannot be explained simply by thermal expansion, and the ^13^C chemical shifts as observed by solid-state NMR are different for all sites, not just those exhibiting disorder. Moreover, the presented crystal structure prediction procedure that does not consider disorder in an output form described both experimental **TFM** polymorphs in the energetic global minimum region. In conclusion, our study shows the power of combining experiment, namely DSC, X-ray diffraction, and MAS NMR, with DFT calculation and CSP to probe and understand the solid-state landscape, and in particular the role of dynamics, for pharmaceutical molecules.

## 4 Experimental and Computational Procedures

### 4.1 Synthesis and Crystallization of **TFM**

All investigated compounds were synthesized by the Jinan YSPharma Biotechnology Co. Ltd. (Jinan, China). The purity of the obtained compounds was >98%, as confirmed by HPLC and solution-state NMR (see Figure S3 in the Supporting Information). Crystals of **TFM** were obtained by crystallization from a dichloromethane solution at 5 °C.

### 4.2 Single-Crystal X-ray Diffraction of **TFM**

Single-crystal X-ray diffraction experiments of a single crystal of **TFM** were carried out at 100 and 292 K using an Oxford SuperNova single-crystal diffractometer with microsource Cu Kα radiation (λ = 1.5418 Å) and a Titan detector Oxford Diffraction (Agilent Technologies, Yarnton, U.K.) equipped with a 800 Cryostream low-temperature unit (Oxford Cryosystems, Oxford, U.K.).

Diffraction data collection, cell refinement, data reduction, and absorption correction were performed using the CrysAlis PRO software (Oxford Diffraction). Structures were solved by the direct method SHELXS^102^ and then refined using the full-matrix least-squares method SHELXL 2015^103^ implemented in the OLEX2 package.^104^ In all of the crystal structures, the non-hydrogen atoms were present in the direct-methods solution.

### 4.3 Solid-State NMR Spectroscopy of **TFM**

Cross-polarization magic angle spinning (CP MAS) NMR and one-pulse ^1^H MAS experiments were performed, except where otherwise stated, on a 600 MHz Avance III spectrometer, operating at 600.13, 564.68, 150.90, and 60.81 MHz for ^1^H, ^19^F, ^13^C, and ^15^N, respectively, equipped with a HX MAS probe head using 4, 2.5, and 1.3 mm ZrO_2_ rotors. Except where otherwise stated, a recycle delay of 45 s was used. Additional ^1^H → ^13^C CP MAS NMR measurements were performed on a Bruker Neo spectrometer operating at 850.23 and 213.73 MHz for ^1^H and ^13^C, respectively, equipped with an HXY probe operating in double-resonance mode using 4 mm ZrO_2_ rotors. A recycle delay of 60 s was used.

A sample of U-^13^C,^15^N-labeled histidine hydrochloride was used to set the Hartmann–Hahn conditions for ^13^C and ^15^N. ^1^H → ^13^C and ^1^H → ^15^N CP MAS experiments on the 600 MHz Avance III spectrometer were performed at a MAS frequency of 12 kHz with a proton 90° pulse length of 4 μs and contact times of 2 ms for ^13^C and 8 ms for ^15^N. For cross-polarization, the nutation frequency was 50 kHz for ^13^C as well as ^15^N for with a ^1^H ramp shape from 90% to 100% with a ^1^H nutation frequency of 62.5 kHz. For ^13^C and ^15^N, 3.5k and 2k data points were acquired for spectral widths of 40 and 28 kHz, respectively. ^1^H → ^13^C CP MAS experiments on a 850 MHz Bruker Neo spectrometer were performed at a MAS frequency of 12 kHz with a proton 90° pulse length of 3.5 μs and a contact time of 2 ms. For cross-polarization, the nutation frequency was 71 kHz for ^13^C with a ^1^H ramp shape from 90% to 100% with a ^1^H nutation frequency of 83 kHz. A total of 4k data points were acquired for a spectral width of 58.8 kHz. In all cases, a SPINAL-64 decoupling sequence^105^ with a ^1^H nutation frequency of 71.4 kHz and pulse length of 7 μs was applied (also for the PISEMA experiment described below). For recording of the ^19^F →^13^C CP MAS NMR spectra at a MAS frequency of 28 kHz, a proton 90° pulse length of 2.5 μs, and a contact time of 2 ms with a ^1^H ramp shape from 90% to 100% with ^19^F and ^13^C nutation frequencies of 75 and 47 kHz, respectively, were used. A total of 3.5k data points were recorded for a spectral width of 62.5 kHz. The acquisition data were collected with a decoupling sequence containing one 180° pulse per rotation period.^106^ In all CP experiments, the following phase cycling was employed: ^1^H excitation pulse *y–y*, ^1^H contact pulse *x*, ^13^C contact pulse *x*, *x*, −*x*, −*x*, *y*, *y*, −*y*, −*y*, with receiver phase *x*, −*x*, −*x*, *x*, *y*, −*y*, −*y*, *y*. A ^19^F single-pulse MAS spectrum was acquired with a ^19^F 90° pulse length of 2.5 μs, a spectral width of 250 kHz, and a time domain size of 1k data points. As a sample to setup ^19^F experiments, polytetrafluoroethylene (PTFE) was used.

The PISEMA MAS experiment^83,87,107^ was carried out with an ^1^H nutation frequency of 82.5 kHz in all of the experiments, and the ^13^C spin-lock field strengths were adjusted to the first-order sideband condition, ω_13C_ = ω_1H_ ± ω_r_. The spinning frequency was 13 kHz and was regulated to ±3 Hz by a pneumatic control unit. A total of 96 transients were coadded for each of 64 *t*_1_ FIDs, corresponding to a total experimental time at 77 h. The 2D PISEMA MAS experiments incremented the SEMA (spin exchange at the magic angle) contact time using a step of 16.28 μs, with a maximum *t*_1_ evolution time of approximately 1 ms. The phase cycling was as follows: ^1^H excitation pulse *y–y*, ^1^H magic angle pulse −*y*, ^1^H contact pulse −*x*, ^13^C contact pulse *x*, ^1^H SEMA pulse *x*, ^13^C SEMA pulse *x*, receiver *x*, −*x*. Only cosine-modulated data were collected. Thus, a real Fourier transformation was performed on the *t*_1_ data that yielded spectra with a symmetrized ω_1_ dimension and dipolar splitting. Since the *t*_1_ time signal increases with increasing SEMA contact time, the ω_1_ dimension was processed using the baseline correction mode “qfil” in the Bruker TopSpin 3.5 program software,^108^ which subtracted a constant intensity from the time signals prior to the Fourier transformation and yielded spectra free from the dominant zero-frequency peak that gives the ^1^H–^13^C doublet.

Fast MAS spectra were recorded with a spin rate of 62.5 kHz. The ^13^C–^1^H invHETCOR (for indirect detection of ^13^C) experiments were performed using the pulse sequence described by Mao et al.^109^ The following parameters were used: a proton 90° pulse length of 2.5 μs, a first contact time of 2 ms, a second contact time of 1 ms or 150 μs, both with a ^1^H ramp shape from 90% to 100%. The ^1^H and ^13^C nutation frequencies were 160 and 101 kHz, respectively, for both CP steps. The acquisition data were collected with a SWf-TPPM^110,111^ decoupling sequence with a ^1^H nutation frequency of 10 kHz and a pulse length of 50 μs.^105^ The phase cycling was as follows: ^1^H excitation pulse *x*, ^1^H first contact time pulse *y*, ^13^C first contact pulse *x*, first ^13^C pulse in z-filter block *y*, first ^1^H suppression of ^12^C magnetization pulse *x*, second ^1^H suppression of ^12^C magnetization pulse *y*, second ^13^C pulse in the z-filter block *y*, – *y*, ^1^H second contact pulse *x*, ^13^C second contact pulse *x*, receiver *x*, −*x*. The maximum evolution times were *t*_1max_ = 4.2 ms and *t*_2max_ = 20 ms, with 8 coadded transients averaged for each of 180 *t*_1_ FIDs, corresponding to a total experimental time of 24 h. The ^1^H–^1^H DQ-SQ experiment was performed with one rotor period of BaBa recoupling^76−78^ at a ^1^H nutation frequency of 160 kHz. A 16-step phase cycle was used to select Δ*p* = ±2 on the DQ excitation block and Δ*p* = −1 on the final 90° pulse, where *p* is the coherence order. The maximum evolution times were *t*_1max_ 1.0 ms and *t*_2max_ 17.2 ms, with 16 coadded transients averaged for each of 128 *t*_1_ FIDs, corresponding to a total experimental time of 42 h. The States-TPPI method was employed for sign discrimination.^112^

Adamantane (resonances at 38.48 and 29.46 ppm) was used as a secondary ^13^C chemical-shift reference from external tetramethylsilane (TMS) in all experiments.^113,114^ The ^15^N chemical shift was referenced indirectly to neat liquid ammonia by using powdered ^15^*N*-glycine as an external secondary reference at δ 34.40 ppm.^114,115^ The ^19^F chemical shift was referenced indirectly to CCl_3_F by using PTFE as an external secondary standard at δ −122.7 ppm.^114^ It is well-known that the real temperature inside the MAS rotor depends on numerous factors, mostly related to frictional effects caused by rotor spinning.^116^ In this work, Pb(NO_3_)_2_ as a commonly accepted solid-state NMR thermometer^117^ was used for temperature calibration.

### 4.4 QM Calculations

DFT calculations were performed with periodic boundary conditions using the CASTEP 19.11 code.^57^ The geometry optimizations were performed until the energy converged to within 10^–7^ eV using the X-ray diffraction crystal structures as an input file. For all calculations, the generalized density approximation DFT functional PBE with the MBD* dispersion correction scheme (DFT-D method) was applied,^118,119^ and the maximum plane wave cutoff energy was 630 eV using an ultrasoft pseudopotential.^120^ A comparison of the average forces remaining on the atoms after geometry optimization was carried out, varying all atoms with the unit cell parameters fixed or varying all atoms and the unit cell parameters (the convergence limit was 0.03 eV/Å). We observed average forces (given as Cartesian components) up to ca. 0.02 eV/Å. The energetic barrier calculations through changes in the C4–NH–C5–C6 torsional angle were carried out by using the DFT-D method for the full periodic system, though with the crystal lattice symmetry reduced to *P*1. All atomic coordinates were allowed to relax except for the selected torsional angle and unit cell parameters. In all cases, the optimization algorithm was BFSG^121^ and the Monkhorst–Pack grid^122^ of minimum sample spacing 0.07 × 2π Å^–1^ was used to sample the Brillouin zone. The NMR chemical shifts were computed using the gauge including projected augmented wave (GIPAW) method.^55,56^ The calculated NMR chemical shieldings were transformed to chemical shifts by linear regression between calculated and experimental results.

### 4.5 CSP Calculations

The molecule was first geometry minimized in the gas phase at the PBEPBE/6-311G(d,p) level of theory using Gaussian16,^123^ and flexible torsions were determined through second derivatives and finite difference perturbations. We also took into account chemical intuition and the fact that computational time increases significantly when the number of degrees of freedom increases. Local approximation models (LAMs) were therefore constructed for the **TFM** molecule, treating the two torsional angles describing possible rotation of the aromatic ring as well as the CF_3_ group as independent degrees of freedom. LAMs were constructed using a uniform grid along the one-dimensional degrees of freedom, at 30° increments. The global search was performed using CrystalPredictor II^124^ employing a smoothed intramolecular potential,^125^ with 500000 minimizations in the *Z′* = 1 investigation and 1000000 in the *Z′* = 2 investigation. Dispersion–repulsion contributions toward the lattice energy were estimated by using a Buckingham exp-6 function with the potential parameters for C, H–C (hydrogen attached to carbon), polar hydrogen, N, O, and F.^126−130^ Following analysis and clustering, CrystalOptimizer^131^ was used to refine the 1000 lowest energy structures in each investigation, at the same level of theory, with additional flexibility introduced (angles around torsions treated as flexible in the global search). The lattice energies reported are given per formula unit.

### 4.6 Other Methods (DSC, Elemental Analysis)

Differential scanning calorimetry (DSC) was recorded using a Mettler-Toledo DSC 3 with a heating/cooling rate of 5 °C min^–1^.

Elemental analyses of hydrogen, carbon, and nitrogen were performed using CE Instruments.

## Supporting Information Available

.magres files for GIPAW calculations (ZIP)
Further discussion of solid-state NMR spectra, experimental X-ray powder pattern calculated diffractograms based on the experimental X-ray single crystal structures, solution-state ^1^H NMR spectrum, for the geometry optimization, unit cell parameters, densities and energies, and the hydrogen-bonding arrangements, GIPAW-calculated shieldings, crystal structure prediction, and the CASTEP code used during fixed torsion angle calculations (PDF)

## Accession Codes

CCDC 1892916 and 1969989 contain the supplementary crystallographic data for this paper. These data can be obtained free of charge via www.ccdc.cam.ac.uk/data_request/cif, or by emailing data_request@ccdc.cam.ac.uk, or by contacting The Cambridge Crystallographic Data Centre, 12 Union Road, Cambridge CB2 1EZ, UK; fax: +44 1223 336033.
