# Supplementary material for: Synergy of Solid-State NMR, Single-Crystal X-ray Diffraction, and Crystal Structure Prediction Methods: A Case Study of Teriflunomide (TFM)
Source: Cryst Growth Des. 2021 May 10;21(6):3328–43. doi: 10.1021/acs.cgd.1c00123 (PMC8273857; doi:10.1021/acs.cgd.1c00123)
Supplement: Supplementary file 2 — cg1c00123_si_002.pdf [file cg1c00123_si_002.pdf]

SUPPORTING INFORMATION

Synergy of Solid-state NMR, Single Crystal X-Ray Diffraction and Crystal Structure Prediction methods – A case study of Teriflunomide (TFM).

Tomasz Pawlak,[1] Isaac Sudgen,[2] Grzegorz Bujacz,[3] Dinu Iuga,[4] Steven P. Brown,[4] Marek J. Potrzebowski[1]

[1] Centre of Molecular and Macromolecular Studies, Polish Academy of Sciences, Sienkiewicza 112, 90-363 Lodz, Poland.

[2] Molecular Systems Engineering Group, Centre for Process Systems Engineering, Department of Chemical Engineering, Imperial College London, London SW7 2AZ, UK

[3] Institute of Molecular and Industrial Biotechnology, Lodz University of Technology, Stefanowskiego 4/10,, 90-924, Lodz, Poland

[4] Department of Physics, University of Warwick, Coventry, CV4 7AL, UK

SUPPLEMENTARY TO THE DISCUSSION OF RESULTS

S1) Discussion about Spinning Sidebands displayed in Figure 5 and S4.

The difference seen when we compare the spectra shown in Figure 5 and S4(a,b) to that in Figure 2a is the intensity and number of spinning sidebands: spinning sidebands (denoted by asterisks) are numerous and intense and more evident in Figure 5 and S4(a,b). This is to be expected since the experiments displayed on Figure 5 and S4(a,b) were performed at a 20 T magnetic field (^{1}H Larmor frequency of 850.2 MHz), while the room temperature measurements in Figure 2a were carried out a 14 T (^{1}H Larmor frequency of 600.1 MHz) and Chemical Shift Anisotropy (CSA) is proportional to the strength of the magnetic field. In addition, the averaging of the CSA by dynamics, in particular for the phenyl resonances given the discussion of temperature-dependent intensity changes in Figure 3, needs to be considered. From an inspection of the spinning-sideband patterns at −80 ^{0}C for the phenyl resonances in Figure 5b and S4b and comparison with simulated (using the Topspin software[1]) sideband patterns for the CSA values calculated using the GIPAW methodology in Figure S2c, for the low temperature polymorph (Z' = 2), it appears that the phenyl ring is rigid and does not undergo reorientation.

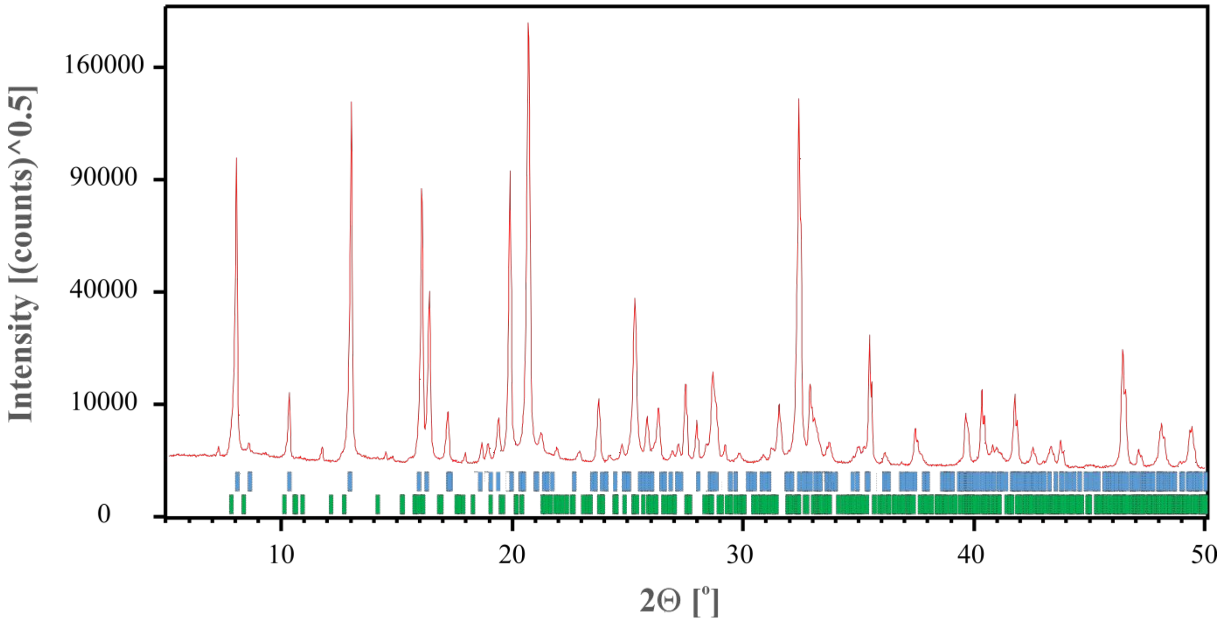

Figure S1. X-Ray powder pattern for **TFM** material crystallized from dichloromethane. Recorded at 295 K on a Rigaku SmartLab 3 kW system in Bragg Brentano geometry and with a Cu Kα (λ = 1.5425 Å) source. The total collection time was 100 min. The calculated peak positions are indicated for the X-ray single crystal structure of **TFM**^{RT} (blue) and **TFM**^{LT} (green).

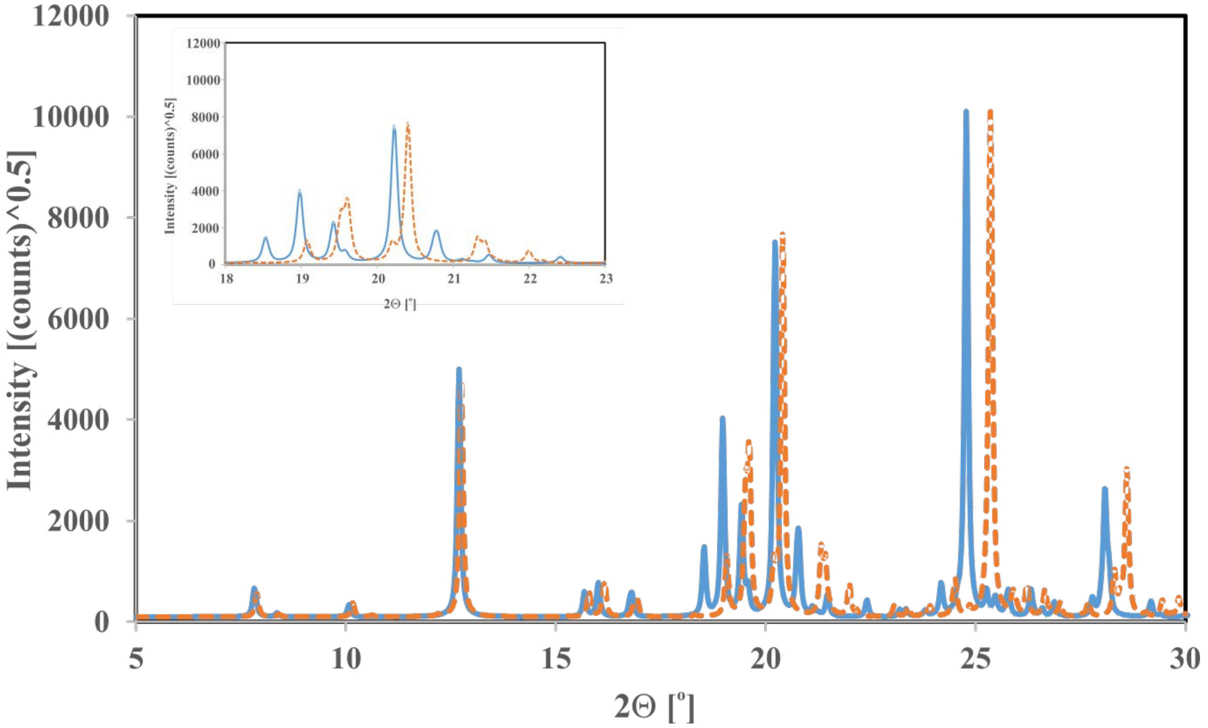

Figure S2. Calculated diffractogram based on the X-ray single crystal structure of **TFM**^{RT} (blue) and **TFM**^{LT} (dotted orange).

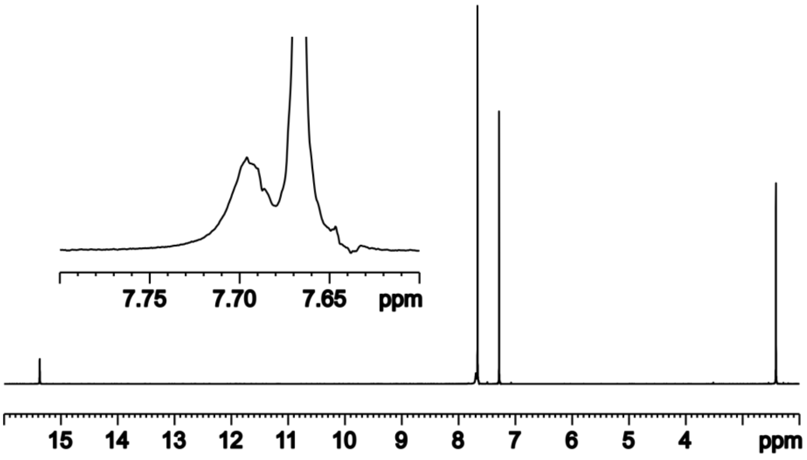

Figure S3. Solution-state ^{1}H NMR ($CDCl_3$) of **TFM**. The chemical shifts are as follows: CH_3 = 2.24 ppm, 6-H = 7.66 ppm, 7-H = 7.28 ppm, NH = 7.69 ppm, OH = 15.37 ppm.

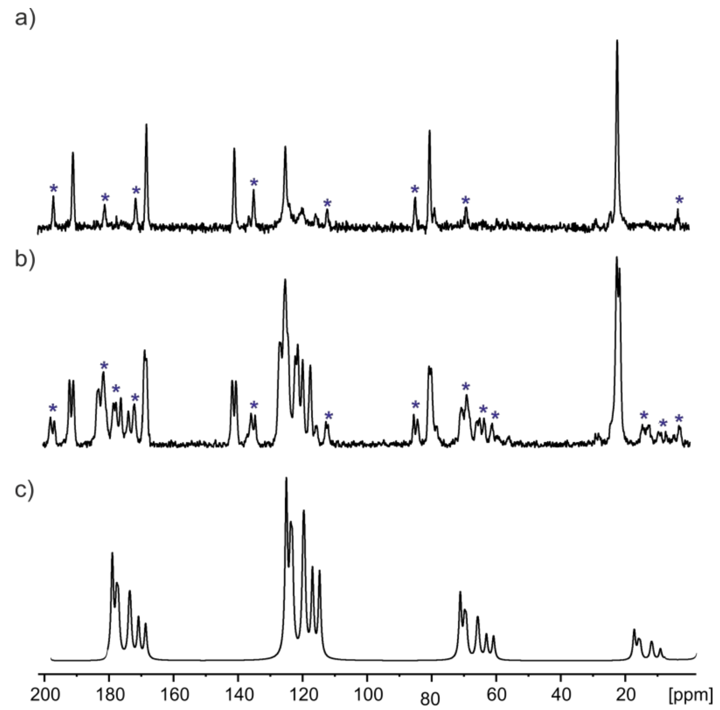

Figure S4. $^1H \rightarrow ^{13}C$ CP MAS NMR spectra of **TFM** recorded at a spinning rate of 12 kHz at a) 20 °C and b) –80 °C and a ^1H Larmor frequency of 850.2 MHz. 48 and 64 transients were co-added for a recycle delay of 60 s for (a) and (b), respectively. Asterisks indicate spinning sidebands. c) Line shape ^{13}C spectrum simulated using the Topspin software for the aromatic resonances of the **TFM^{LT}** structure based on the calculated GIPAW chemical shifts and chemical shift anisotropy and a line broadening of 200 Hz.

Table S1. Unit cell parameters for the single crystal X-Ray diffraction structures of **TFM** and as obtained after DFT-D (CASTEP) geometry optimisation

**Parameter**	**X-Ray data**		**DFT-D (opt all)[a]**		**DFT-D (opt all + cell)[b]**	
	**TFM^{LT}**	**TFM^{RT}**	**TFM^{LT}**	**TFM^{RT}**	**TFM^{LT}**	**TFM^{RT} A/B conformer[c]**
**Unit cell parameters**						
**a [Å]**	9.4030(2)	4.85221(15)	9.403	4.852	9.639	4.867/ 5.037
**b [Å]**	11.5300(2)	10.8738(2)	11.530	10.873	11.294	10.739/ 9.801
**c [Å]**	11.9572(3)	11.7015(3)	11.957	11.701	12.628	11.303/ 11.899
**α [°]**	95.9600(17)	102.454(2)	95.96	102.45	95.804	101.36/ 104.28
**β [°]**	105.8499(19)	97.821(2)	105.85	97.821	112.13	99.880/ 94.436
**γ [°]**	110.763(2)	93.832(2)	110.76	93.832	111.22	93.202/ 90.223
**Density of crystal [g/cm^3]**	1.579	1.510	1.579	1.510	1.572	1.579/1.582

[a] DFT-D geometry optimization of all atomic positions with the unit cell parameters fixed to those determined by single-crystal X-ray diffraction
[b] DFT-D geometry optimization of all atomic position and allowing the unit cell parameters to vary
[c] The DFT calculations of the A and B conformer of **TFM^{RT}** were made for two separate systems where the starting unit cell contains the experimental A or B conformer only. This leads to different unit cell parameters.

Table S2. Total lattice energy difference (compared to the lowest energy) for **TFM** after DFT-D (CASTEP) geometry optimisation.

Structure	Total lattice energy difference [kJ/mol][a]
**TFM^{LT} (opt all)**	0.09
**TFM^{LT} (opt all + cell)**	0
**TFM^{RT} A occupancy sites (opt all)**	2.29
**TFM^{RT} A occupancy sites(opt all + cell)**	1.58
**TFM^{RT} B occupancy sites (opt all)**	2.39
**TFM^{RT} B occupancy sites (opt all + cell)**	0.75

[a] The lowest energy structure in the table was assigned to an energy of 0.

Table S3. The NH...N intermolecular and OH...O intramolecular hydrogen-bond arrangements in **TFM** for the single crystal X-Ray diffraction structures and as obtained after DFT-D (CASTEP) geometry optimisation

Structure		X...Y distance [Å]		H...Y distance [Å]		XHY angle [°]	
		N…N	O...O	H…N	H…O	NHN	OHO
**TFM^{LT}** (molecule A)	X-Ray data	3.112	2.481	2.298	1.742	158.0	148.9
	DFT-D (opt all)[a]	3.025	2.445	2.019	1.455	164.0	154.1
	DFT-D (opt all + cell)[b]	2.991	2.442	1.984	1.449	164.4	154.1
**TFM^{LT}** (molecule B)	X-Ray data	3.052	2.501	2.239	1.763	148.8	157.6
	DFT-D (opt all)[a]	2.979	2.455	1.970	1.468	164.8	154.0
	DFT-D (opt all + cell)[b]	2.942	2.453	1.929	1.465	164.9	153.9
**TFM^{RT}** (lower A occupancy sites)	X-Ray data	3.131	2.491	2.314	1.755	158.7	148.4
	DFT-D (opt all)[a,c]	2.982	2.462	1.971	1.467	165.4	153.8
	DFT-D (opt all + cell)[b,c]	2.955	2.454	1.943	1.461	165.8	154.6
**TFM^{RT}** (higher B occupancy sites)	X-Ray data	3.131	2.491	2.314	1.755	158.7	148.4
	DFT-D (opt all)[a,c]	2.980	2.459	1.970	1.468	165.2	154.4
	DFT-D (opt all + cell)[b,c]	2.969	2.448	1.964	1.452	165.8	154.9

[a] DFT-D geometry optimization of all atomic positions with the unit cell parameters fixed to those determined by X-ray diffraction
[b] DFT-D geometry optimization of all atomic position and allowing the unit cell parameters to vary

[c] The DFT calculations of A and B occupancy sites of **TFM^{RT}** were performed for two separate systems where the starting unit cell contain the experimental A or B sites only.

Table S4. GIPAW calculated ^{13}C and ^{1}H NMR nuclear shieldings, σ [in ppm], for **TFM^{RT}** and **TFM^{LT}** (after DFT-D geometry optimization allowing the unit cell to vary).

Structure (position)	σ(^{1}H)				σ(^{13}C)			
	**TFM^{RT}** (lower A occupancy	**TFM^{RT}** (higher B occupancy	**TFM^{LT}** (molecule A)	**TFM^{LT}** (molecule B)	**TFM^{RT}** (lower A occupancy	**TFM^{RT}** (higher B occupancy	**TFM^{LT}** (molecule A)	**TFM^{LT}** (molecule B)
**1**	28.3[a]	27.8[a]	28.3[a]	27.8[a]	150.9	148.1	149.7	148.6
**2**	---	---	---	---	-28.4	-28.2	-26.9	-26.5
**3**	---	---	---	---	91.2	89.3	90.8	89.7
**4**	---	---	---	---	2.3	1.0	1.0	2.2
**5**	---	---	---	---	28.4	26.2	26.7	28.4
**6**	23.9	23.4	24.4	23.4	51.1	50.4	53.7	48.8
**6'**	23.5	22.5	22.4	23.8	51.0	49.8	51.1	48.3
**7**	23.7	22.6	23.1	22.5	43.8	43.2	43.4	44.0
**7'**	23.9	23.1	24.6	22.9	43.0	41.2	41.8	42.1
**8**	---	---	---	---	47.7	46.0	47.7	45.2
**9**	---	---	---	---	31.0	29.7	29.8	30.1
**10**	---	---	---	---	48.9	48.4	48.9	48.5
**-NH-**	20.6	20.5	20.8	20.4	---	---	---	---
**-OH**	13.1	12.9	12.8	13.0	---	---	---	---

[a] The average of the distinct ^{1}H chemical shifts is presented.

Table S5. An analysis of the 10 lowest-energy structures obtained for CSP of **TFM** with Z' = 1 and after DFT-D geometry optimisation, including comparison with the DFT-D optimised structure of **TFM^{RT}**.

After DFT-D (CASTEP) optimisation				CSP output				
Energy ranking	Energy difference (kJ/mol)[a]	Similarity[b]	RMSD$_{15}$[c]	Energy ranking	CSP Energy difference (kJ/mol)[a]	Similarity[b]	RMSD$_{15}$[c]	Space group
1	0.00	13 out of 15	0.886	7	2.04	13 out of 15	0.934	C2/c
**2**	**0.03**	**15 out of 15**	**0.276**	**1**	**0.00**	**15 out of 15**	**0.387**	**P-1**
3	0.10	15 out of 15	0.624	4	1.51	15 out of 15	0.388	P-1
4	0.19	7 out of 15	0.388	9	2.43	7 out of 15	0.393	P-1
5	0.71	8 out of 15	1.068	6	1.93	9 out of 15	2.652	P21/c
6	2.37	7 out of 15	0.466	8	2.19	10 out of 15	1.460	C2/c
7	2.43	9 out of 15	0.537	10	2.52	11 out of 15	0.271	P-1

8	3.05	7 out of 15	1.443	5	1.88	7 out of 15	0.413	P-1
9	3.58	9 out of 15	0.807	3	1.21	7 out of 15	0.629	P21/c
10	4.43	12 out of 15	0.961	2	0.24	13 out of 15	1.165	P21/c

The structure best matched to the experimental X-ray single-crystal diffraction structure is shown in bold.
[a] Relative to the lowest energy structure at CSP or CASTEP level.
[b] Determined using the COMPACK crystal structure similarity procedure implemented in the CCDC Mercury software.
[c] The average root-mean squared difference over all similar molecules determined using the CCDC Mercury software for clusters of molecules built up from the experimental X-ray single-crystal diffraction and calculated structures.

Table S6. An analysis of the 10 lowest-energy structures obtained for CSP of **TFM** with Z' = 2 and after DFT-D geometry optimisation, including comparison with the DFT-D optimised structure of **TFM**$^{\mathbf{LT}}$.

**After DFT-D (CASTEP) optimisation**				**CSP output**				
**Energy ranking**	**Energy difference (kJ/mol)**[a]	**Similarity**[b]	**RMSD$_{15}$**[c]	**Energy ranking**	**CSP Energy difference (kJ/mol)**[a]	**Similarity**[b]	**RMSD$_{15}$**[c]	**Space group**
**1**	**0.00**	**15 out of 15**	**0.222**	**4**	**0.94**	**15 out of 15**	**0.387**	**P-1**
2	0.87	15 out of 15	0.341	2	0.67	15 out of 15	0.463	P-1
3	3.27	15 out of 15	0.494	3	0.93	15 out of 15	0.569	P21/c
4	4.41	12 out of 15	0.531	1	0.00	11 out of 15	1.327	P-1
5	4.44	11 out of 15	1.141	5	1.13	11 out of 15	1.568	P-1
6	4.78	15 out of 15	0.592	9	1.92	15 out of 15	0.611	P-1
7	5.35	11 out of 15	0.535	8	1.66	11 out of 15	0.670	P21/c
8	10.13	9 out of 15	0.643	7	1.53	9 out of 15	0.533	P-1
9	14.24	9 out of 15	1.434	6	1.49	14 out of 15	0.664	P-1
10	17.50	7 out of 15	0.596	10	3.20	15 out of 15	0.385	P-1

The structure best matched to the experimental X-ray single-crystal diffraction structure.
[a] Relative to the lowest energy structure at CSP or CASTEP level.
[b] Determined using the COMPACK crystal structure similarity procedure implemented in the CCDC Mercury software.
[c] The average root-mean squared difference over all similar molecules determined using the CCDC Mercury software for clusters of molecules built up from the experimental X-ray single-crystal diffraction and calculated structures.

Note that the DFT geometry optimizations for energetic barrier calculations were performed with fixed torsion angles using the following code in the CASTEP .cell file:

```
%BLOCK NONLINEAR_CONSTRAINTS
torsion C 15 0 0 0 N 3 0 0 0 C 13 0 0 0 C 21 0 0 0
torsion C  3 0 0 0 N 1 0 0 0 C  1 0 0 0 C  9 0 0 0
%ENDBLOCK NONLINEAR_CONSTRAINTS
```

REFERENCES

(1) *Topspin, Version 3.5, Bruker Biospin Gmbh, Karlsruhe, Germany.*

(2) Bradley, J. P.; Tripon, C.; Filip, C.; Brown, S. P. Determining Relative Proton–Proton Proximities from the Build-up of Two-Dimensional Correlation Peaks in ^{1}H Double-Quantum MAS NMR: Insight from Multi-Spin Density-Matrix Simulations. *Phys. Chem. Chem. Phys.* **2009**, *11* (32), 6941–6952. https://doi.org/10.1039/B906400A.

(3) Brown, S. P. Probing Proton–Proton Proximities in the Solid State. *Prog. Nucl. Magn. Reson. Spectrosc.* **2007**, *50* (4), 199–251. https://doi.org/10.1016/j.pnmrs.2006.10.002.

(4) Brown, S. P. Applications of High-Resolution ^{1}H Solid-State NMR. *Solid State Nucl. Magn. Reson.* **2012**, *41*, 1–27. https://doi.org/10.1016/j.ssnmr.2011.11.006.
